# Analysis of monocyte cell tractions in 2.5D reveals mesoscale mechanics of podosomes during substrate-indenting cell protrusion

**DOI:** 10.1242/jcs.259042

**Published:** 2022-05-27

**Authors:** Hendrik Schürmann, Fatemeh Abbasi, Antonella Russo, Arne D. Hofemeier, Matthias Brandt, Johannes Roth, Thomas Vogl, Timo Betz

**Affiliations:** 1 Institute of Cell Biology, ZMBE, University of Münster, Von-Esmarch-Straße 56, D-48149 Münster, Germany; 2 Third Physical Institute, University of Göttingen, Friedrich-Hund-Platz 1, D-37077 Göttingen, Germany; 3 Institute of Immunology, University of Münster, Röntgenstraße 21, D-48149 Münster, Germany

**Keywords:** Podosome, Traction force microscopy, Mesoscale, Actin, Protrusion, Buckling

## Abstract

Podosomes are mechanosensitive protrusive actin structures that are prominent in myeloid cells, and they have been linked to vascular extravasation. Recent studies have suggested that podosomes are hierarchically organized and have coordinated dynamics on the cell scale, which implies that the local force generation by single podosomes can be different from their global combined action. Complementary to previous studies focusing on individual podosomes, here we investigated the cell-wide force generation of podosome-bearing ER-Hoxb8 monocytes. We found that the occurrence of focal tractions accompanied by a cell-wide substrate indentation cannot be explained by summing the forces of single podosomes. Instead, our findings suggest that superimposed contraction on the cell scale gives rise to a buckling mechanism that can explain the measured cell-scale indentation. Specifically, the actomyosin network contraction causes peripheral in-plane substrate tractions, while the accumulated internal stress results in out-of-plane deformation in the central cell region via a buckling instability, producing the cell-scale indentation. Hence, we propose that contraction of the actomyosin network, which connects the podosomes, leads to a substrate indentation that acts in addition to the protrusion forces of individual podosomes.

This article has an associated First Person interview with the first author of the paper.

## Abstract (precis)

**Summary:** Using a buckling model, we extend the current description of local podosome protrusion and include a mechanical explanation for protrusion on the cell scale.

## INTRODUCTION

Breaching physical barriers is an essential step in leukocyte extravasation (Nourshargh et al., 2010) as well as cancer metastasis (Reymond et al., 2013). To help cells perform this function, podosomes in healthy cells (and invadopodia in invasive cancer cells) intrinsically combine matrix metalloproteinase (MMP)-mediated degradation (Ferrari et al., 2019; Wiesner et al., 2013) and F-actin-driven protrusion (Bouissou et al., 2017; Revach et al., 2015). Despite functional similarities, the distinct biological roles of podosomes and invadopodia (Paterson and Courtneidge, 2018) mean that researchers must clearly differentiate between the two in order to successfully develop drugs that target invadopodia rather than podosomes. Over the past two decades, multiple differences have been highlighted, with the most important being related to podosome organization and their collective dynamic behavior (Destaing et al., 2003; Meddens et al., 2016). With the introduction of mechanobiology into the field of podosome biology, the understanding of single podosome behavior has advanced greatly, as mechanical and mechanosensory activity can now be assigned to these structures (Labernadie et al., 2014; van den Dries et al., 2019b). However, these detailed biomechanical studies have tended to focus on force generation of individual podosomes. While podosome interconnectivity has been linked to mesoscale dynamics (Meddens et al., 2016; Proag et al., 2015), details of the link between podosome mechanical connectivity and single podosome mechanics remain largely unknown. Likewise, little is understood about the possible collective effects of podosomes that might act together to provide protrusion capabilities on the whole-cell level.

Podosomes occur in various (super)structures beyond the characteristic punctate actin protrusions. Individual podosomes are frequently arranged in variously sized and crowded clusters. Podosome numbers have been shown to depend on substrate stiffness (Labernadie et al., 2014). At lower resolution, the fluorescence signals obtained from fluorescently labeled podosomal actin appear as an interpodosomal ‘cloud’ (Destaing et al., 2003), whereas at higher resolution, podosomes have a fine-tuned nano-architecture. Furthermore, researchers have found that an architectural correlate of mesoscale connectivity exists in the connecting cable network that spans between adjacent podosomes (van den Dries et al., 2019a). Although connectivity has previously been assumed to occur via passive mechanical coupling (Proag et al., 2015), emerging evidence of a myosin IIA-dependent dynamic role suggests that active forces and tension may build up within the interconnecting actomyosin network (Meddens et al., 2016).

In general, such contractile forces of the actomyosin network are known to play a major role in cellular force generation and motility (Cai et al., 2006; Kraning-Rush et al., 2011). Regarding the substrate-facing podosome actin network, three major elements can be distinguished: the characteristic F-actin-rich core, the myosin IIA-decorated F-actin-based lateral filaments, and the dorsal interpodosomal filaments (van den Dries et al., 2019a). As assumed based on composition, researchers studying single podosomes have found that the myosin IIA-decorated lateral filaments are responsible for generating protrusion forces (Van Den Dries et al., 2013). However, a corresponding active mechanical role for the densely myosin IIA-decorated interpodosomal actin network has yet to be identified.

It has been reported that when podosome-bearing cells are seeded on soft substrates, the cells display large-scale out-of-plane deformations (Kronenberg et al., 2017; Linder, 2007). Because they have predominantly focused on force generation by single podosomes, previous studies have adopted analysis methods to spatially filter out such cell-scale deformations in order to avoid interfering with the localized pushing of individual podosomes (Kronenberg et al., 2017; Proag et al., 2015). This approach has resulted in high-resolution force analyses that have enormously advanced the understanding of single podosome mechanics; however, the mechanical implications on the whole-cell level have remained unaddressed.

In this study, we explore the cell-scale traction force exerted by cells on a compliant 2D hydrogel using traction force microscopy (TFM) (Butler et al., 2002). In contrast to most previous studies, we assess the vertical force along with planar forces; this approach is typically referred to as 2.5D TFM. Complementing previous studies, we focus on larger-scale (i.e. cell-wide) out-of-plane deformations. To minimize biological variance, we employ murine Lifeact–EGFP-labeled ER-Hoxb8-derived monocytes (Riedl et al., 2008). These cells represent a recently established estradiol-dependent conditionally immortalized model of myeloid progenitors (Wang et al., 2006). Previously, these cells have been used successfully in cell migration studies and have been shown to be comparable to standard primary cells with respect to podosome formation and degradation (Accarias et al., 2020).

We demonstrate that differentiated ER-Hoxb8 monocytes regularly display widely scattered podosomes upon treatment with lipopolysaccharide (LPS), an endotoxin of Gram-negative bacteria that is capable of triggering inflammation (Płóciennikowska et al., 2015; Raetz et al., 1991). After analyzing traction stresses (i.e. externally transmitted forces), we analyze the correlation between traction force foci and actin punctae that represent podosomes. Although we observe that traction foci can be associated with actin punctae, not all actin punctae are associated with force generation. Additionally, the focal planar forces are accompanied by a broad indentation into the compliant gels. Analyzing the resolved tractions and out-of-plane deformation with respect to their geometry, we propose a scale-dependent spatiotemporal model of podosome mechanics. Here, network contraction is assumed to cause planar tractions, while buckling instability acts as the main driver for substrate-indenting protrusion.

## RESULTS

### Surface *z*-plane extraction allows for simple, fast 2.5D TFM and ventral actin projection

**Fig. 1.**
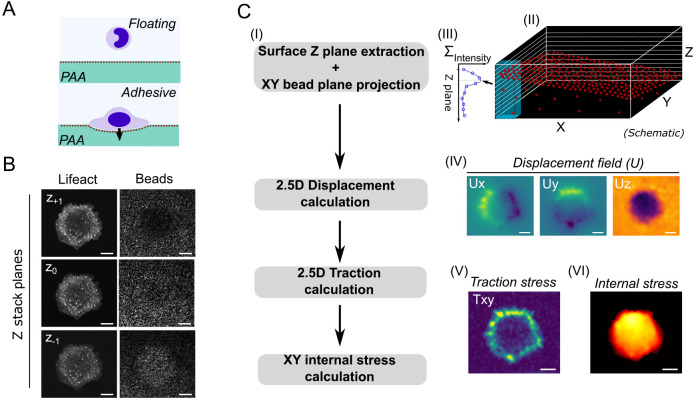
***In vitro* modeling and analysis of 2.5D mechanics in monocytes.** (A) Illustration of out-of-plane deformation and indentation caused by adherent cells on soft hydrogels. (B) Multiplane image stack of a representative LPS-treated, Lifeact–EGFP-labeled ER-Hoxb8 monocyte, showing the Lifeact signal and fluorescent beads in the substrate. The adjacent *z* planes (0.5 µm plane distance) reveal ventral actin punctae and a broad indentation beneath the cell body. Images are representative of 29 cells. (C) The workflow for mechanical analyses is shown in I. The surface coordinates of the gels (*z*; shown in the schematic in II) were extracted by finding the peak coordinate of a spline interpolation (III) to the local intensity profile along *z* (as highlighted in the schematic). The peak coordinate is indicated by an arrow and dotted line in III. IV: repetitively computing the surface coordinates and subtracting the unloaded reference map yielded the *z* deformation map (U*z*). Together with U*x* and U*y*, which were calculated from the 2D bead projections after applying the respective *z* surface maps, the 2.5D tractions were calculated using established algorithms. From the in-plane tractions (T*xy*, shown in V), the internally relayed normal (i.e. compressive) stress (shown in VI) could be deduced using an established finite element method. The shown deformation and stress maps were computed from the cell shown in B. Images in IV, V and VI are color-coded to show centripetal Ux and Uy in yellow–blue, substrate indentation in purple (IV), absolute traction spots in yellow–green (V), and the internal stress distribution in yellow–red (VI). Scale bars: 10 µm.

To gain deeper insights into the mechanics of cell protrusion, a full and dynamic capture of in-plane and out-of-plane forces is essential. As such, we used 3D spinning-disk image stacks to show the relevance of substrate-indenting forces. While the actin structures as measured by Lifeact–EGFP were visible in several planes penetrating inside the substrate, the fluorescent beads marking the substrate surface left the focal plane in the indented regions (Fig. 1A,B); these were then used to determine the *z* position of the substrate surface. While state-of-the-art free-form deformation analysis (Jorge-Peñas et al., 2015) was used to readily determine the *xy*, hence, in-plane components of the deformation field, we assessed the local *z* displacement using a different approach that employed a grid with 1.6 µm distance resolution. For each grid point, the fluorescence intensity of individual *z* planes was analyzed along the *z* direction. Expressing the resulting fluorescence intensity function using spline interpolation (Fig. 1C II, III) allowed us to define the surface position with an accuracy of ∼0.1 µm (Fig. S1F). This analysis was performed for each grid point, resulting in a height profile of the hydrogel surface. As illustrated in Fig. 1C II, the surfaces were, in general, not oriented perfectly parallel to the image plane, so we corrected for this by subtracting the fit of a plane that represents this error. Comparing the surface topology between the situation with and without cells allowed us to infer the *z* deformation field (U*z*; Fig. 1C IV*).* Quantifying both the *xy* and the *z* deformation of the surface enabled us to perform 2.5D TFM for the ER-Hoxb8 monocytes. Using fundamental momentum conservation approaches, we were then able to determine the cell-internal stress transmission from the in-plane traction forces (T*xy*; Fig. 1C V, VI).

The lateral force resolution of TFM has been determined previously and is on the order of micrometers (Sabass et al., 2008). To determine the resolution in our measurements, we simulated 3D bead stacks (Fig. S1) based on established methods (Jorge-Peñas et al., 2015). This allowed us to estimate the lateral (*xy*) traction force resolution of 2 µm for a point force with a radius in the range of podosome radii (0.9 µm) and a traction magnitude of 100 Pa (Fig. S1D). This lateral force resolution is, thus, consistent with the literature, whereas the lateral resolution of the *z* forces is about a factor of two lower. This means that podosome force centers separated by a distance smaller than 4 µm would be observed as a single indentation center (Fig. S1E). Of note, we focused on out-of-plane deformation (U*z*) instead of force (T*z*) throughout the study because of its biological relevance in this context. While the force along *z* represents the cause, the deformation is then the observed effect of indentation.

Beyond 2.5D TFM, applying ‘2.5D imaging’ of the intracellular actin dynamics provides a convenient add-on. Specifically, by applying the *z* coordinates of the surface map to the Lifeact–EGFP channel, we were able to project the substrate-facing, ventral cell surface into a single plane. This results in neither a maximum projection, which would overlay the full actin signal of the cell, nor a single image plane. Instead, this method specifically extracts the actin signal along the surface of the deformed substrate and, thus, depends on the *z* plane distance. These projected images allowed us to easily compare the traction forces and actin concentrations close to the substrate.

The presented 2.5D TFM approach comes with an in-plane traction resolution of 2 µm and the capacity to extract the actin signal above the substrate despite the substrate deformation. This combination allows for detailed cell-scale mechanical analyses in substrate-indenting cells.

### Broad indentations are accompanied by local in-plane tractions at podosomes

**Fig. 2.**
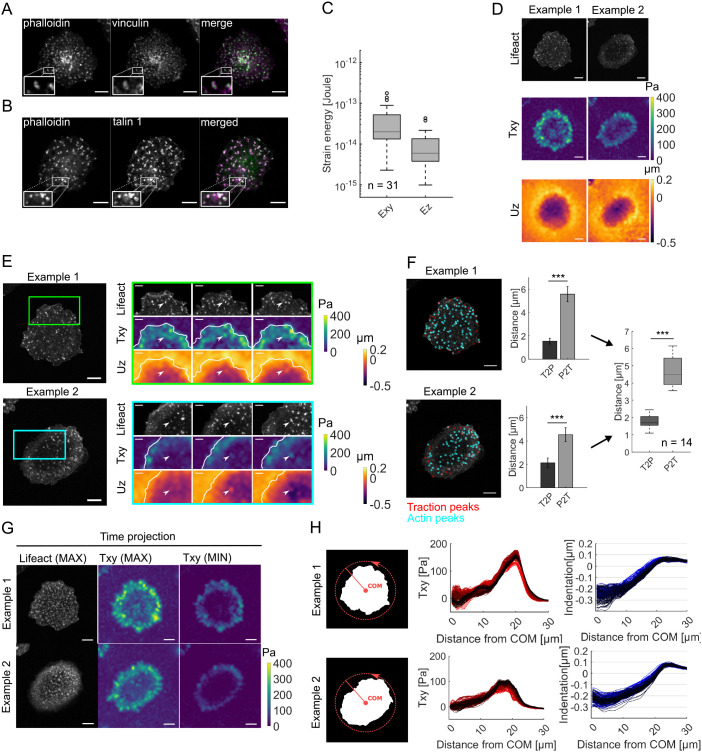
**Podosome-forming monocytes exert cell-wide tractions that can be one-sidedly correlated to podosomes in the presence of broad indentations.** (A,B) LPS-treated ER-Hoxb8 monocytes were seeded on soft PAA gels, fixed, and then stained for (A) vinculin and (B) talin-1, in addition to labeling of F-actin using fluorescent phalloidin. Vinculin and talin-1 partially colocalize with and partially form rings around phalloidin-stained actin punctae, defining them best as podosomes. In merge images, vinculin and talin-1 are shown in magenta, and F-actin is shown in green. Images are representative of 12 and 9 cells for vinculin and talin-1, respectively. (C) A mechanical analysis of scattered actin punctae-displaying cells revealed a substantial strain energy expenditure along the *z* direction (E*z*) compared to that in the *xy* direction (*Exy*). E*z*/E*xy*=0.29±0.16 (mean±s.d., *n*=31 cells from three independent experiments). (D) A common mechanical pattern was found across the cells. While tractions mostly aligned at the cell boundary, the indenting area spanned over the cell area. Representative images show F-actin, T*xy* and U*z* for two example cells out of 29. (E) In multiple cells, the focally resolved tractions and their dynamics could be correlated to U*z* and podosomes. Boxes in the example F-actin images (left) denote regions shown on the right. Cell outlines are marked in the T*xy* and U*z* maps, and arrowheads denote the location of apparent actin reorganization. Images are representative of 14 cells. (F) Quantifying the mutual *k*-nearest neighbor distance between a traction (red) and podosome actin peaks (blue) exposed smaller traction-to-podosome distances (T2P) than podosome-to-traction distances (P2T) over the measured time series (bar graphs show mean±s.d. of 188 frames for two representative cells out of 14). Across these cells, a one-sided colocalization for T2P (mean±s.d.: 1.8±0.4 µm) in contrast to P2T (mean±s.d.: 4.6±0.8 µm) could be shown (boxplot, *n*=14 cells from three independent experiments). (G) A key feature of the temporal evolution could be observed across the majority of cells: whereas actin scattered widely and apparently stochastically over the entire cell area, in-plane traction appeared and disappeared in the center while being robust at the periphery (images show the indicated time projections of F-actin and T*xy* for two example cells, representative of *n*=24 cells from three independent experiments). (H) Plotting the in-plane tractions and out-of-plane deformation as a function of distance from the cell COM revealed a circularly symmetrical centripetal traction ring at the periphery (plotted in red, middle) and a central broad indentation (plotted in blue, right). Darker colors represent later time frames; *n*=200 frames. Diagrams (left) illustrate circular projections by rotation around the COM for one frame each. ***P<0.001 (Mann–Whitney U-test). Boxplots in C and F show the 25th and 75th quantiles (box) in addition to the median value (line). The whiskers extend to 1.5 times the interquartile range beyond the 25th and 75th quantile, with points outside this plotted separately. Scale bars: 10 µm in A,B,D–G; 5 µm in E insets.

LPS-treated ER-Hoxb8 monocytes were seeded on soft polyacrylamide (PAA) gels (3 kPa), and the seeded cells displayed scattered actin punctae on their ventral side. Immunofluorescence staining for vinculin (Fig. 2A) and talin-1 (Fig. 2B) revealed a close relation to actin. Although most signals colocalized with actin, some were arranged in rings around the actin punctae (Fig. 2A,B, insets). Therefore, this signaling best characterizes the actin punctae as podosomes. The absence of a general core–ring pattern, which is characteristic of podosomes, can be attributed to the reduced imaging capacities on the soft hydrogel setup.

A main result of our analysis is that we observed a broad out-of-plane deformation for cells, with podosomes distributed all over the cell area. To understand the origin of this large-scale indentation, we first quantified the ratio between out-of-plane (E*z*) and in-plane (E*xy*) strain energy, and obtained a ratio of E*z*/E*xy=*0.29±0.16 (mean±s.d., *n*=31), highlighting that a substantial proportion of the deformation energy generated by the cell was being expended in the *z* direction (Fig. 2C). Focusing on the spatial distribution, common patterns could be observed for in-plane tractions (T*xy*) and substrate indentation (U*z*) (Fig. 2D–H). Traction foci arose throughout the cell-covered area but were pronounced as a ring at the cell periphery (Movie 1). In contrast, the accompanying substrate protrusion (U*z*) spanned over the entire cell area, with the strongest indentation at the cell center (Fig. 2D). Although the lateral resolution to separate individual indentations was limited to 4 µm, this does not explain the large-scale indentation at the central region, as there was no observable increase in podosome density in that region.

Although this large-scale indentation seems, at first sight, at odds with previous reports that describe a local indentation at the single podosome scale (Kronenberg et al., 2017; Proag et al., 2015), here we did not filter out large-scale indentation as was done in previous studies to obtain better contrast of single podosome forces. Still, we regularly observed local elevations of U*z* above the background noise (Fig. 2D,E; Movie 2). In multiple cells with sharply resolved, dynamic podosome clusters in the 2D projections (*n*=14), detailed analysis of the peripheral traction region revealed a series of traction hot spots. Based on the size and dynamics, these hot spots might represent not just single traction centers, but also multiple traction centers that are beyond the resolution limit of the deformation detection. The traction force centers arose at sites of podosomes and colocalized with the local U*z* elevations, in particular at sites of apparent podosome reorganization (Fig. 2E, arrowheads).

We next wondered whether a similar correlation between prominent traction foci and podosomes was also observable throughout the cell; however, we observed no mutual colocalization between traction hot spots and podosomes, as shown in Fig. 2F.

To analyze possible correlations between traction force centers and actin punctae that represent podosomes, we quantified the distance between them. This was done in two ways. First, we measured, for each traction peak, the distance to the next podosome (T2P). To only include real and cell-scale prominent traction peaks, percentile-based thresholds were empirically defined with respect to the intrinsic background noise levels (see Materials and Methods). Then, we looked at the distance from every podosome to the next traction force peak (P2T). It should be noted that these two quantities do not have to be equal. Quantification revealed a distance of 1.8±0.4 µm for T2P, in contrast to 4.6±0.8 µm for P2T distances (mean±s.d.; *n*=14; *P*<0.001, Mann–Whitney U-test), suggesting that many podosomes were not generating notable in-plane traction forces. It should be noted that this does not contradict previous reports on single podosome indentation forces, as we focus here on the lateral traction.

These results suggest that although a force spot requires a close podosome, not all podosomes generate traction forces. It is important to note that the main lateral traction forces are generated at the cell periphery, which is consistent with findings in other cell types (Legant et al., 2013).

### Planar tractions comprise two spatiotemporally distinct components

Taking these surprising but very preliminary observations together with the absence of previous reports on cell-wide traction generation via podosomes or its relation to single podosome mechanics, we wondered whether cell-wide indentation may be an additional support structure to the reported local indentations of podosomes. To further evaluate the potential role of podosomes, we next performed a general characterization of cell-wide mechanics. To this end, we focused on cells showing cell-wide scattered actin punctae. We analyzed 200 frames per cell with a capture interval of 5 s, which accumulated to around 16 min of recording time per cell.

To obtain a broad picture of the spatiotemporal pattern of deformation and traction (Fig. 2G,H), we chose two simple approaches: first, we looked at the temporal evolution of in-plane traction force centers; and second, we studied the spatial mechanical pattern over time. To visually overlay the forces and the actin signal over time, we calculated a simple maximum projection of the actin signal and the traction forces, T*xy*, over the entire time series*.* To obtain a further visual representation of the force changes within this interval, we also generated a minimum projection for T*xy* (Fig. 2G). Comparing maximum and minimum projections allowed us to assess the spread of force during the observation time. Surprisingly, the minimum and maximum traction projections taken together revealed two distinct traction components – a peripheral ring-like traction and focal, transient tractions that emerged over the entire cluster area. By contrast, the maximum time projection of the Lifeact signal showed no correlative pattern but displayed a ventral cell surface with densely, homogeneously scattered podosomes in the majority of cells (*n*=24).

Motivated by the circular structure displayed by the force patterns, we wondered how the centripetal force component changes as a function of distance to the cell center. To study this, we averaged the centripetal force around the cell for each radius and plotted the radial force as a function of the distance to the cell center of mass (COM). This was repeated for the cellular indentation into the substrate, U*z* (Fig. 2H). Although we lost tangential information along the circumference, we obtained profiles that quantified cell-wide mechanical properties under the symmetry displayed by the system. For instance, the radial projection of T*xy* yielded the traction component acting toward the cell center as a function of distance from the cell center (0.54 µm bin width). Positive traction components indicated centripetal, negative centrifugal orientation, while the smoothness of the curve and the spread of the data provided information about the degree of circular symmetry. As illustrated for the two example cells, the indentations presented as rotationally symmetrical, shallow and smoothly curved protrusions (Fig. 2H, blue) that began descending close to a peripheral, centripetal traction ring (Fig. 2H, red). In summary, in-plane traction forces, which are stable over time, are found at the cell periphery, whereas localized and short-lived lateral traction peaks are found in the central region of the cell.

### Two mechanical patterns characterize podosome cluster mechanics

All forces obey Newton's laws of momentum conservation. In the case of mechanical traction force transmission during substrate adhesion, this manifests within the cell in the form of force balance. In many cell types, force balance is ensured by stress fibers that span across the cell and the nucleus to connect the focal adhesions (Schwarz and Gardel, 2012). However, in the absence of such stress fibers, other structures have to be in place to ensure force equilibrium inside the cell. To determine the intracellular stress components, we used a finite element approach that allowed us to derive the stress field inside our cells (internal stress), as previously demonstrated for other systems (Tambe et al., 2011).

**Fig. 3.**
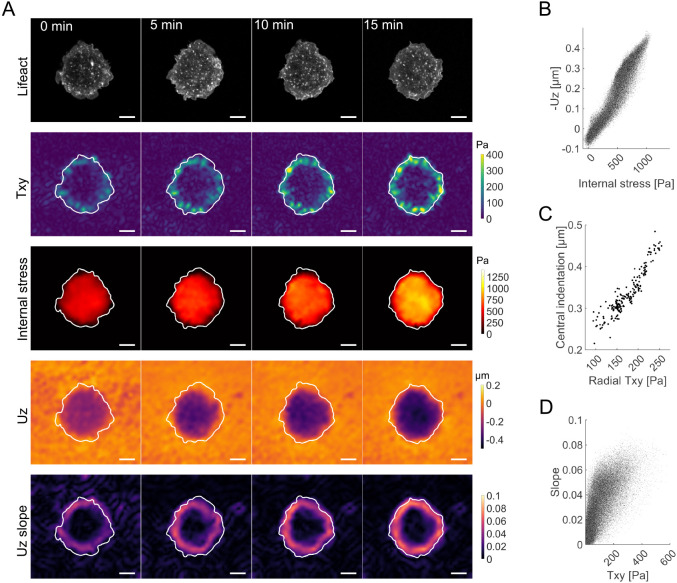
**Full mechanical analysis of an example podosome-forming monocyte.** (A) The dynamic actin pattern (first row) is accompanied by a stable circular planar traction ring, which displays a dynamic magnitude (T*xy*, second row). The resulting stress builds up inside the podosome-covered area (third row) associated with a similarly shaped out-of-plane deformation (U*z*, fourth row). The *z* deformation exposes a peripherally aligned U*z* slope (fifth row). Cell outlines are marked in the second to fifth rows. Cell shown is representative of *n*=29 cells that are compared in Fig. 4B. Scale bars: 10 µm. (B) The internal normal stress due to T*xy* locally correlates with U*z* (Pearson coefficient of 0.95; calculated pixelwise over 200 time frames). (C) This is accompanied by a strong correlation between the radial T*xy* and the central indentation yielded from the circular projection profile (Pearson coefficient of 0.93; calculated over 200 time frames). (D) Finally, the U*z* slope shows an elevated correlation with T*xy* (Pearson coefficient of 0.73; pixelwise calculated over 200 time frames). For reasons of illustration, only every 250th value is depicted in the local correlation plots in B and D. The correlation coefficients were calculated over all 200 frames (capturing interval: 5 s).

**Fig. 4.**
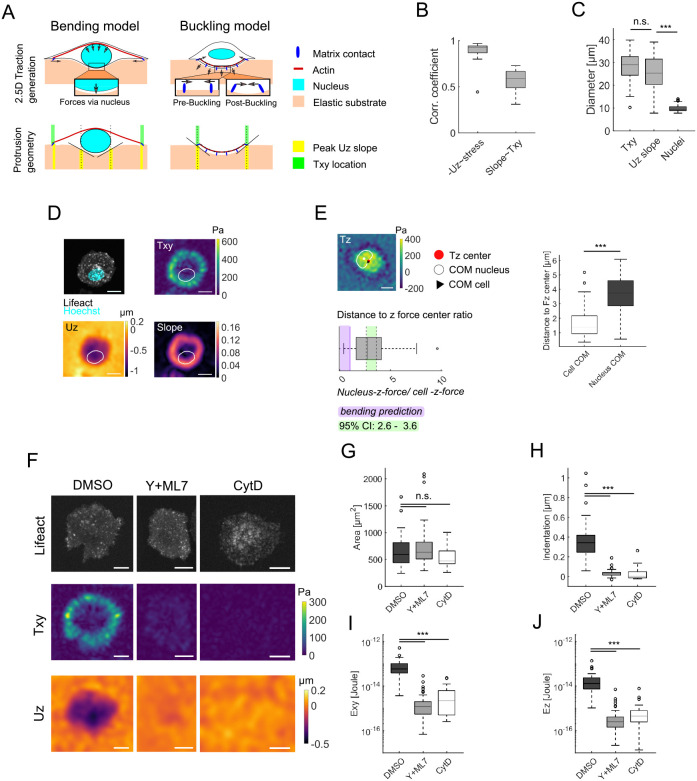
**Two opposing mechanical models for out-of-plane traction generation tested against each other for plausibility.** (A) Schematic illustration of nuclear bending and network buckling. The bending model considers indentation to be caused by the nucleus pushing into the substrate due to redirected forces from distant tractions (green). The distant tractions can create small rotational moments (peripheral yellow); however, the indentation defining maximal indentation slope (peak U*z* slope) is restricted to the nuclear size (central yellow). In contrast, network buckling is nucleus independent, and indentation occurs right between the exerted tractions due to network compression. Planar tractions (green) and the indentation shape defining peak U*z* slope (yellow) colocalize. (B) A pixel-wise correlation between U*z* and internal stress (left) showed a strong correlation, with a Pearson correlation coefficient of 0.89±0.10 (mean±s.d.) as possibly explained by both models. However, the U*z* slope also moderately correlates with T*xy* (right; Pearson correlation coefficient of 0.57±0.11, mean±s.d.), which can be more easily explained by the buckling model (*n*=29 cells and 200 time points each, from three independent experiments). (C) With regards to indentation geometry, as expected by their correlation, the mean diameter that is spanned between the radially projected T*xy* and U*z* slope are of comparable length (28±8 µm and 25±9 µm, respectively; mean±s.d., *n*=29; n.s., not significant, *P* =0.18, Mann–Whitney U-test), whereas the mean nuclear axis length in LPS-treated monocytes is only 10±1 µm (mean±s.d., *n*=66 cells; ***P<0.001 compared with peak U*z* slope diameter, Mann–Whitney U-test). This makes a predominant role of the nucleus unlikely. (D) Representative images showing the relation between nuclei (stained using Hoechst 33342; cyan in fluorescence image, outlined in maps), T*xy*, U*z* and U*z* slope. (E) Although the exclusive indentation analysis is a good hint, final conclusions can only be drawn by directly testing the mechanically proposed causes: bending requires the *z* force center to colocalize with the nuclear COM, whereas buckling requires the *z* force center to colocalize with the cell COM (as shown in the T*z* image, top left). By quantifying the absolute distances between the respective COM to the *z* force center (F*z*) in cells with a mean indentation of more than 0.1 µm beneath the nucleus (right; *n*=64 cells from four independent experiments), we find a significantly closer relation between the cell COM to *z* force center across all cells (***P<0.001, Mann–Whitney U-test). Comparing the distance ratios across all cells (bottom left) yields 3.1±0.3 (mean±s.e.m.; *P*<0.001, two-tailed one-sample *t*-test; 95% confidence interval, 2.6–3.6) and rejects the bending hypothesis. (F–J) The structural component underlying the buckling is likely to be found in actomyosin. (F) Cells treated with Y-27632 and ML-7 (Y+ML7; *n*=59 cells from three independent experiments) or with cytochalasin D (CD; *n*=28 cells and 3 gels from two independent experiments) exert substantially smaller tractions and do not indent into the substrate, whereas DMSO-treated cells do (*n*=41 cells from four independent experiments). While CD treatment changes the podosome F-actin phenotype, cells treated with Y+ML7 have a phenotype comparable to that of control cells. (G) Y+ML7- and CD-treated cells show no differences in cell mask area compared to DMSO-treated control cells (*P*=0.20 and 0.08, respectively). (H) Y+ML7- and CD-treated cells lose indentation almost entirely, which is accompanied by strong decreases in (I) in-plane (E*xy*) and (J) out-of-plane (E*z*) strain energy by more than an order of magnitude. In G–J, ***P<0.001; ns, not significant (Mann–Whitney U-test). Boxplots show the 25th and 75th quantiles (box) in addition to the median value (line). The whiskers extend to 1.5 times the interquartile range beyond the 25th and 75th quantile, with points outside this plotted separately. Scale bars: 10 µm.

The resulting stress tensor is a complex property that can be separated into different subcomponents. Of special interest here is the normal component of the internal stress, which reflects the tensional stress. In our case, the internal stress quantifies the contractile force generated by the actomyosin network within the cell and between the podosomes. Guided by our preliminary mechanical characterization, we additionally included the indentation slope (U*z* slope) to quantify the location and area of substrate indentation. While the substrate indentation (Fig. 2H) quantifies the overall deformation, the slope measures the gradient and, hence, the local variability of the indentation. The respective analyses resulted in patterns like the one shown in Fig. 3A, and further summarized across all cells in Fig. 4B,C.

Again, the 5 s acquisition interval exposed a highly dynamic punctate actin signal that was evenly distributed on the ventral cell surface (Fig. 3A, first row). In contrast, the mechanical patterns, although displaying common dynamics in magnitude, did not show any substantial changes in topography. Instead, two distinct shapes characterizing the results stood out most. The first shape was a peripheral, circular pattern exhibited by the planar traction component (Fig. 3A, second row), which was also shown by the indentation slope (Fig. 3A, fifth row). The second was a central pattern that emerged over and covered the podosome area, as displayed by the internal stress (Fig. 3A, third row) and U*z* (Fig. 3A, fourth row). Quantifying this visual relation by the respective Pearson correlation over all time frames (*n*=200) suggested a possible interdependence. In detail, this analysis showed a high correlation between U*z* and the internal stress with a Pearson coefficient of 0.95 (Fig. 3B). A similar high correlation of 0.93 was found between the radial centripetal traction and the indentation at the center of the cell, which were derived from the respective circular projections (Fig. 3C). Furthermore, an elevated correlation of 0.73 was found between the U*z* slope and T*xy* (Fig. 3D). Notably, although the patterns show clear interdependency, the relation is non-trivial, as the *xy*- and *z*-based information were calculated independently. It should be noted that dynamic lateral and indentation force centers in the inner part of the cell can be attributed to previously described and well-known local forces generated by podosomes. However, the cell-wide indentation as observed here seems to be an additional effect that can be explained as a global cell-level effect.

Briefly, the in-plane stress measurements of in-plane traction force and internal stress are found to correlate with the out-of-plane deformation measures (U*z*, U*z* slope). This suggests a mechanical relation between these generally independent quantities.

### Deformation and traction geometry suggest a buckling-induced indentation mechanism

The correlation between internal stress and out out-of-plane deformation can generally be achieved by one of two opposing mechanical mechanisms: redirected bending or buckling. Redirected bending, as the ‘classical’ view, considers the forces to be relayed from peripheral contact points (here, supposedly podosomes) to the cortex or stress fibers and then intracellularly redirected onto the nucleus, resulting in a nuclear-driven pushing into the substrate. Hence, the intracellular stress is transmitted along the top of the cells, thereby pushing the intracellular material down. As only the nucleus has a reasonable stiffness, it will transmit these forces onto the substrate (Fig. 4A, left). In contrast, buckling considers the internal stress as located on the ventral plane, and the vertical deformation is driven by an instability due to actomyosin network contraction. In this view, the internal stress transmission and the main substrate deformation are aligned perpendicularly (Fig. 4A, right).

In principle, both models can explain the strong correlation found here of 0.89±0.10 (mean±s.d., *n*=29) between internal stress and deformation for indentation depths greater than 0.1 µm (Fig. 4B). However, differences between the models arise due to indentation and force geometry, namely between the position of the nucleus and the indentation. According to the bending model, substrate indentation occurs at the site of nuclear indentation. Consequently, the peak indentation (U*z*) slope (Fig. 4A, yellow) is expected to be in the range of and limited by nuclear size. In contrast, the buckling model predicts the highest slope to be close to or colocalizing with the underlying planar tractions (Fig. 4A, green).

After calculating the local Pearson correlation coefficients between T*xy* and the U*z* slope for indentation depths greater than our detection limit of 0.1 µm, we found an elevated correlation of 0.57±0.11 (mean±s.d., *n*=29; Fig. 4B). For further analyses, we defined the averaged traction (T*xy*) and peak U*z* slope diameters from the circular projections. To this end, the distance between the peak value of the respective projection and the COM was multiplied by two. In a next step, the T*xy* and peak U*z* slope diameters were compared to the mean nuclear axis lengths in LPS-treated and nuclear-stained monocytes (Fig. 4C). The two diameters were derived from the coordinates of the peak of the respective circular projections, and thus represent the mechanically active area. Consistent with the correlation coefficients, the T*xy* and peak U*z* slope diameters were 28±8 µm and 25±9 µm (mean±s.d.), respectively (*n*=29, *P*=0.18, Mann–Whitney U-test). However, the mean nuclear axis measured was substantially smaller at ∼10±1 µm (mean±s.d., *n=*66, *P*<0.001, Mann–Whitney U-test), which is in strong contrast to the predictions of the nuclear indentation model (Fig. 4A,D).

Conclusive evidence in favor of buckling over bending cannot be reached by this simple geometrical consideration. Therefore, we next addressed the underlying physical cause of indentation, which would serve to exclude one of the models. While the bending model requires the *z* force center (i.e. the weighted center of T*z*) to overlap with the nuclear center, buckling is based on the *z* force center overlapping with the center of the cell. We thus analyzed the nuclear-stained indented cells with respect to their substrate-indenting *z* force (threshold 50 Pa), as well as their nuclear and cell centers, in cells with a mean indentation greater than 0.1 µm beneath the nucleus (*n*=64) (Fig. 4E). We found that the general distribution revealed a substantially lower distance between the *z* force and cell center (1.6±0.1 µm, mean±s.e.m.) compared to the distance between the *z* force and nuclear center (3.7±0.2 µm, mean±s.e.m.; *P*<0.001, Mann–Whitney U-test). This became even more striking when we examined the ratios between these distances for individual cells. Here, we found about a threefold greater distance between the *z* force center and the nuclear center as compared to the distance between the *z* force center to the cell center (3.1±0.3, mean±s.e.m.). Taken together, these findings suggest that the bending model is not responsible for the surface indentation; the buckling model is a much more likely mechanism.

### Indentation is mediated by actomyosin

Having identified buckling as the rational mechanism of indentation, we still lacked further evidence on whether this is mediated by interpodosomal actomyosin. To this end, we performed perturbation experiments to compare tractions and indentation depths after manipulating the actomyosin network. This was done by treating cells with Y-27632 and ML-7 (Y+ML7), which inhibits contraction via ROCK1 and/or ROCK2 and myosin light chain kinase (MLCK), or by adding cytochalasin D (CD), which compromises actin polymerization (Fig. 4F–J). Whereas DMSO- and Y+ML7-treated cells showed a comparable podosome appearance, CD altered the phenotype (Fig. 4F). A comparison of the cell mask areas employed for mechanical analyses revealed no significant difference between DMSO (*n*=41) and Y+ML7 (*n*=59; *P*=0.21, Mann–Whitney U-test) or DMSO and CD (*n*=28; *P*=0.08, Mann–Whitney U-test) treatment, excluding a major area effect for strain energy calculation (Fig. 4G). However, we did observe losses in indentation for Y+ML7-treated (0.03±0.03 µm, mean±s.d.) as well as CD-treated (0.03±0.06 µm, mean±s.d.) cells compared to DMSO-treated controls (0.36±0.21 µm, mean±s.d.) (Fig. 4H), and these losses were accompanied by decreases in *xy* (Fig. 4I) and *z* strain energies (Fig. 4J) by more than an order of magnitude (*P*<0.001 each, Mann–Whitney U-test). These results confirm a key role for actomyosin in indentation, and, together with the above experiments, suggest that buckling of actomyosin is the cause for indentation. This is demonstrated in Fig. S2, where we plot the indentation as function of the internal stress. While the bending model predicts a linear increase here, the buckling model requires a threshold intracellular tension to be established before indenting the substrate, as this is energetically more favorable. Indeed, in spontaneously indenting cells (Fig. S2E,F) and in cells where the indentation was pharmacologically reduced by the addition of CD (Fig. S2H,I), we saw that the indentation was only relevant above intracellular tension values on the order of 100 Pa (Fig. S2G,J).

In conclusion, our results indicate that actomyosin-driven buckling causes broad substrate indentations in podosome-bearing monocytes. Linking individual traction spots to podosomes in a one-sided relation, we provide evidence that the podosome clusters generate global in-plane tractions. Therefore, the broad, cell-wide out-of-plane deformation is also suggested to be caused by cluster effects.

## DISCUSSION

**Fig. 5.**
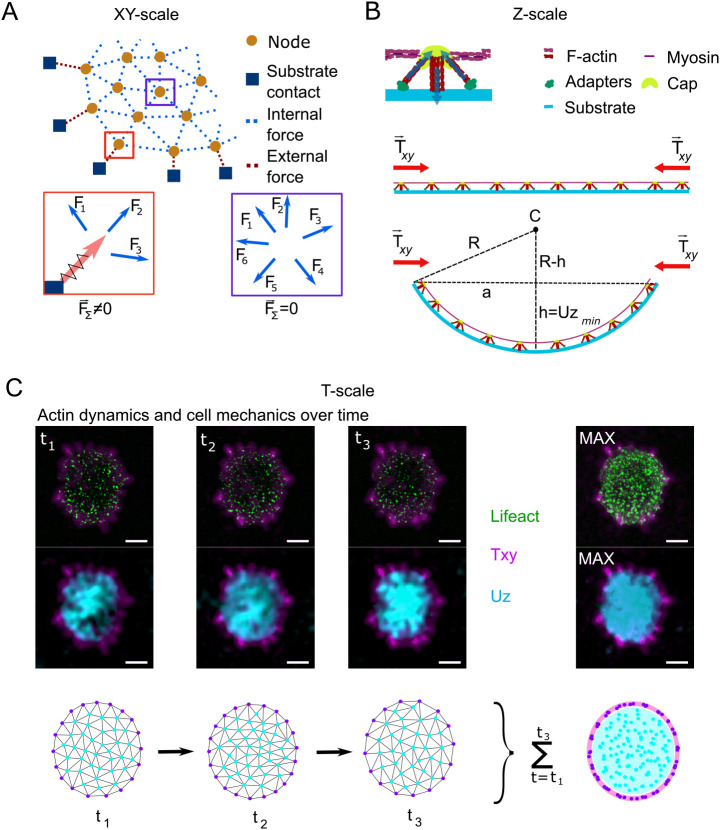
**Proposed *XY–Z–T* model of podosome mesoscale mechanics.** From our data, we propose a model of podosome mechanics on the mesoscale that is mediated by the dorsal actomyosin network. (A) Interpodosomal actomyosin generates contractile forces that are either relayed to neighboring podosomes as internal stress (blue dotted line) or transmitted to the substrate as tractions by podosomes that are not omnidirectionally coupled (red dotted line). Red and purple boxes indicate location-dependent podosome mechanics, shown in greater detail below (F, force). (B) Upon contraction, the substrate connection of the podosome network leads to an increasing internal stress that eventually results in a buckling event of the cell interface towards the substrate right after reaching the instability threshold. *R*, curvature radius; *C*, curvature center; *h*, maximal indentation (minimal U*z*, U*z*_min_); *a*, *Txy* radius. (C) Because the generated tractions are exerted via the most peripheral podosomes that cannot relay forces omnidirectionally, the traction pattern appears stable at the edges of the cell (magenta) despite the dynamically changing node pattern (cyan; first and third row), which finally covers the entire cell area over time (*t*; see maximum time projection, right). Knowing that podosomes locally degrade their substrate, cluster-covering degradation zones (corresponding to the Lifeact signal, green) supposedly colocalize with substrate-indenting protrusion (cyan; second and third row). Time interval from t_1_ to t_3_, 14 min. A rolling ball background subtraction (radius of three pixels) and a median filter (radius of one pixel) were applied to the Lifeact image for better contrast. Scale bars: 10 µm.

Using 2.5D TFM to study podosome- and whole-cell-generated forces in monocytes, we have extended the current view that podosomes only locally exert forces. Our results suggest an integrated lateral force between the podosomes that leads to buckling and subsequent substrate indentation on the cell scale. While we can link long-lasting focal tractions at the cluster border and transient tractions within the cluster to podosomes, we have found that the vast majority of morphologically equal podosomes do not exert substantial in-plane tractions within the resolution of our TFM approach. Another finding is that the observed broad indentation is not explained by the nucleus pushing into the substrate but by the buckling of an as-yet-unknown structure, where the buckling is actomyosin dependent. Assuming that in-plane traction and out-of-plane deformation share the same cause, we propose a mesoscale model of podosome cluster mechanics that can explain our and previous findings by means of the dorsal actomyosin network (Fig. 5).

Forces, in particular protrusion forces, on the single podosome level have been widely investigated over the past decade (Labernadie et al., 2014; Proag et al., 2015; Van Den Dries et al., 2013). In parallel, the complex actin network of the podosome results in the unique formation of superstructures, which have been deciphered to comprise a multilevel organization of F-actin with podosome–substrate and podosome–podosome actomyosin connections (van den Dries et al., 2019a). Although mesoscale connectivity has been shown to play a role in podosome dynamics (Meddens et al., 2016; Proag et al., 2015), no such relation has been shown for force generation on the cell level. Previous studies of podosome mechanics have focused on local protrusions into the substrate, supposedly caused via radial actin fibers in close proximity to the podosomes (Bouissou et al., 2017). However, local protrusions, even if passively coupled, cannot result in the broad indentations that we and others have observed (Kronenberg et al., 2017; Linder, 2007). Instead, in the absence of stress fibers, these indentations are likely the mechanical correlate of podosome mesoscale connectivity.

Basic physical principles suggest that the found traction forces require mechanical contractile stress to be transmitted through the cell. The finding that the region of highest intercellular stress transmission correlated with the regions of largest indentation suggest a causal relation between lateral contraction and indentation into the substrate. The mechanism we propose here to explain these finding is a simple buckling mediated by the high contraction of the actomyosin network that interconnects the podosomes (van den Dries et al., 2019a).

While podosomes at the central region of the cell can balance tensile forces with their neighbors, the fact that podosomes at the cell periphery do not have neighbors in each direction indicates that the stress transmission of the substrate is a contractile traction force (Fig. 5A). Substrate indentation is, then, suggested to occur if the internal stress and the resulting compression within the network surpass a certain threshold (see Materials and Methods), at which the indentation of the substrate is energetically more favorable than the compression (Fig. 5B). Importantly, our ideas do not conflict with the established model of local podosome indentation (Bouissou et al., 2017; van den Dries et al., 2019b) but do extend it to a global extension on the cell scale that is superimposed on the local pushing forces. Due to our method's intrinsic low-pass filter, however, we are unable to resolve these protrusions and can only assume them to be as they have been previously reported (Kronenberg et al., 2017).

In addition to mechanics, our model provides a possible explanation for how degradation and protrusion, two intuitively contrasting processes, can be spatially combined on the cell scale. According to the buckling model, for protrusion to occur, all individual podosomes do not need to touch the substrate if the network is connected to the substrate. If a sufficient number of podosomes adhere to the substrate, others can lose contact due to local degradation without affecting global mechanics. The relatively short lifetime of podosomes therefore leads to a highly dynamic scattering across the cluster that, reportedly, correlates with the observed broad degradation sites (Kremerskothen et al., 2011), whereas the protrusion remains constant and potentially allows podosomes to globally stay in contact with the substrate to form new adhesion and degradation sites (Fig. 5C). This idea, however, is only based on the predictions of our model and requires further investigation.

Our work complements previous studies and may contribute to a deeper understanding of podosome biology and mechanics by relating previous findings. We hope to provide some insights into mechanical forces beyond individual podosomes, which, as we have shown, differ from those of individual podosomes, thus implying scale-dependent mechanics for podosomes, as suggested by the network architecture. Of note, buckling-driven indentation might not be exclusive to podosome-bearing cells. In fact, other types of cells may meet the requirements of having a contractile network that is aligned close to and in parallel to the substrate.

However, some considerations regarding robustness and reproducibility must be made with regard to our cell model and the 2.5D TFM method, and these considerations might explain some inconsistencies between our findings and the literature. Our ER-Hoxb8 cells differentiate into macrophages, whereas many previous studies have investigated dendritic cells, which have been shown to have substantially different behavior, for example upon TLR4 stimulation and integrin expression (Gawden-Bone et al., 2014; Lukácsi et al., 2020). Hence, it is not surprising that LPS does not induce podosome cluster formation in other cells; although, for ER-Hoxb8 monocytes the effect appeared to be robust. Further, inclusion of cells was generously based on Lifeact–EGFP punctae being scattered over the entire cell area. Although we are relatively certain that almost all punctae represent podosomes, as shown by our staining, we cannot determine this for individual punctae without visualization using vinculin or talin-1, which would be interesting for future investigations. An additional aspect originates from the definition of ‘cluster’ used throughout this study; to our knowledge, no well-defined selection criteria for ‘clusters’ exist, so we decided to include cells generously, as mechanical principles should be rather general. We only deviated from this approach for quantifying colocalization between traction foci and podosomes, as this analysis required low noise and sharp cell projections over the entire time series. Although only observed for this selection (14 out of 31 cells), it still served as additional support.

Finally, and importantly, the resolution of our method does not allow study of single podosome mechanics, although we were still able to resolve local events on the mesoscale that manifest at individual podosomes. The approach of performing 2.5D TFM may be helpful for others who study substrate-indenting protrusion on the scale beyond invadosomes and would like to additionally quantify in-plane tractions. However, for invadosomes, complementary methods, such as atomic force microscopy (AFM)- or elastic resonator interference stress microscopy (ERISM)-based methods, have to be employed.

In conclusion, our findings further contribute to the hierarchical (mechano)biology of podosomes by adding mesoscale mechanical aspects. The proposed model relates single podosome mechanics to cluster mechanics. It assigns actomyosin the role of a contractile network that is locally connected to the substrate via individual podosomes. Hence, this contraction allows for in-plane tractions and protrusion forces into the substrate via buckling.

## MATERIALS AND METHODS

### ER-Hoxb8 cell culture

ER-Hoxb8 cell lines were generated as previously described (Wang et al., 2006) from bone marrow cells of Lifeact–EGFP mice. Cell culture and differentiation was performed according to recent work (Heming et al., 2018) with minor adaptations. Briefly, ER-Hoxb8 cells were cultured in uncoated six-well culture plates in RPMI 1640 medium (Merck Millipore, Darmstadt, Germany; Lonza, Basel, Switzerland) supplemented with 10% fetal calf serum (FCS; PAN-Biotech, Aidenbach, Germany; Biowest, Nuaillé, France), 1% L-glutamine (Biochrom, Berlin, Germany), 1% penicillin-streptomycin (Biochrom, Berlin, Germany), 2% GM-CSF (ImmunoTools, Friesoythe, Germany), and 1 µM β-estradiol (Sigma-Aldrich, Steinheim, Germany). Cells were split every 2 days and tested for mycoplasma contamination prior to experiments.

### Differentiation and stimulation of ER-Hoxb8 derived monocytes

ER-Hoxb8 cell suspension was washed and centrifuged twice with phosphate-buffered saline (PBS) containing 10% FCS to remove β-estradiol. Subsequently, 0.5×10^6^–1×10^6^ Lifeact–EGFP-labeled ER-Hoxb8 cells were seeded in 3 ml RPMI 1640 medium supplemented with 10% FCS, 1% L-glutamine, 1% penicillin-streptomycin and 20% supernatant of M-CSF-expressing L-929 cells (ATCC, number CCL-1) in untreated six-well culture plates. Cells were differentiated for 3 days. Non-adherent cells were aspirated and discarded after 2 days. To detach the adherent monocytes, PBS supplemented with 2 mM EDTA (Roth, Karlsruhe, Germany) was used.

For stimulation, differentiation medium (i.e. M-CSF-containing medium) supplemented with LPS (L4391; Sigma-Aldrich, Steinheim, Germany) at a final concentration of 1 µg/ml was added to differentiating cells overnight.

### Traction force experiments

On day 3 after addition of M-CSF-supplemented medium, ER-Hoxb8 cells were detached, centrifuged and resuspended. Subsequently, cells were seeded at numbers of 20,000–25,000 cells per gel (12 mm diameter) and incubated for 30 min. Having removed non-adherent cells, 2 ml differentiation medium supplemented with 25 mM HEPES (Millipore, Burlington, USA) was added. Within the next 1–2 h, 4D stacks were captured at 5 s intervals and 0.5 µm *z* plane distance employing a spinning-disk system (CSU-W1 Yokogawa) in combination with a heating chamber set at 37°C. Podosome-bearing cells included in the study were morphologically defined as displaying scattered actin punctae that did not align into belts or rings. Reference (i.e. null force) images were captured after dropwise addition of 10% sodium dodecyl sulfate (SDS) yielding a final concentration of 0.1%.

For perturbation of myosin-driven contraction or actin polymerization, Y-27632 (688002, Sigma-Aldrich, Steinheim, Germany) and ML-7 (475880, Sigma-Aldrich, Steinheim, Germany) or cytochalasin D (C2618, Sigma-Aldrich, Steinheim, Germany) were added at final concentrations of 20 µM and 10 µM or 2 µM, respectively. DMSO at the highest concentration (0.1%) served as a control. Images for analysis were taken after around 20 min of incubation. The addition of Hoechst 33342 (62249, Thermo Fisher Scientific, Oberhausen, Germany) at a concentration of 0.1 µg/ml 45 min before imaging allowed nuclear visualization for nuclear analysis.

### Gel preparation

If not otherwise stated, all chemicals were purchased from Sigma-Aldrich (Steinheim, Germany). Polyacrylamide (PAA) gels were prepared as previously described (Bollmann et al., 2015) with some modifications. First, glass-bottom dishes (CELLview 35/10 mm, Greiner Bio-One International, Kremsmünster, Austria) were cleaned with 70% ethanol followed by 0.1 N NaOH. Afterwards, the glass bottom was covered with 200 µl (3-aminopropyl)trimethoxysilane (APTMS) for 3 min, thoroughly washed, and then covered with 500 µl 0.5% glutaraldehyde for 30 min. In the meantime, PAA gel pre-mix was prepared by adding 4 µl of acrylic acid to 250 µl of 2% *N,N′-*methylenbisacrylamide and 500 µl of 40% µl acrylamide solution. For 3 kPa-stiff PAA gels, 75 µl of this solution was gently mixed with 415 µl of 65% PBS and 10 µl fluorescent bead solution (100 nm NH_2_-coated micromer-redF; Micromod, Rostock, Germany). Polymerization was induced by addition of 5 µl 10% ammonium persulfate solution (APS) (Roth, Karlsruhe, Germany) and 1.5 µl *N,N,N′,N′*-tetramethylethylenediamine (TEMED) (Roth, Karlsruhe, Germany). Functionalization for coating was performed by activation of the acrylic acid with 0.2 M *N*-(3-dimethylaminopropyl)-*N′*-ethylcarbodiimide hydrochloride (EDC), 0.1 M N-hydroxysuccinimide (NHS), 0.1 M 2-(*N*-morpholino)ethanesulfonic acid (MES) and 0.5 M NaCl for 15 min at room temperature, followed by thorough washing with PBS and incubation with fibronectin (50 μg/ml) and incubation at 37°C for 1 h or at 4°C overnight. The stiffness of the gel was confirmed by rheological measurements (AFM) to ensure consistency.

### *Z* plane extraction from bead planes

For *z* plane extraction of the surface of the hydrogel, the framewise bead channel stacks (11 *z* planes, voxel size 0.108×0.108×0.5 µm) were processed in MATLAB (R2020a, MathWorks Inc., MA, USA). Briefly, we here used columnar-shaped (21×21 pixels×11 planes, except for perturbation experiments, which used 41×41 pixels×31 planes) substacks that laterally shifted over the gel at a predefined overlap (15 pixels, except for perturbation experiments, which used 35 pixels). From these, we repetitively performed a cubic spline interpolation to the signal intensity values along the *z* axis. For interpolation, the increment number was increased by the factor 100. The peak of the spline interpolation was calculated, and the value then assigned to the central *xy* coordinate of the column to fill a discrete matrix prior to cubic interpolation. To project the surface-facing Lifeact–EGFP signal, the surface *z* map was applied to the respective stack to locally project the maximum from up to two planes (i.e. 1 µm) above the extracted plane number. For *xy*-stage drift correction we used dftregistration (Guizar-Sicairos et al., 2008), and as a template randomly chose a 400×400 pixel sized cell-free submatrix. These cell-free regions were also used to determine the *z* distribution (Fig. S1F).

### Traction force and stress calculation

For traction force calculation, the in-plane deformation of the gel was derived from the deviation of the localizations of the fluorescent beads inside the gel at the respective time point from the bead localizations after cell detachment (by SDS) by applying a free-form deformation (FFD) algorithm using the software Elastix (Klein et al., 2010). The calculation was carried out in a three-level pyramid approach from a coarse to a fine scale. Grid size was divided in half and the number of iterations was doubled for each level. The grid size for the finest scale was set to 1.3×1.3 µm or individually up to 1.9×1.9 µm if generating excessive artefacts due to strong tractions. One cell that displayed artifacts using the largest grid was excluded from the study. The final result was reached by optimizing the advanced Mattes mutual information metric using the adaptive stochastic gradient descent algorithm for 2000 iterations at the finest scale. The number of random spatial samples per iteration was chosen based on the size of the respective image. The out-of-plane deformation (U*z*) was calculated from the reference surface *z* map, which was extracted as described above. Prior to inserting into the traction calculation algorithm, the surface *z* map was undeformed by inverse application of the in-plane deformation fields (U*x*, U*y*). U*z* was then calculated by subtraction of the reference surface *z* map from the *xy*-undeformed, force-loaded surface *z* map.

The three-dimensional traction forces exerted by the cells on the gel surface were then inferred from the displacement fields by solving the Tikhonov-regularized equation of the elasticity problem for finite thickness substrates in the Fourier domain (Del Álamo et al., 2007; Jorge-Peñas et al., 2015) using a custom-made MATLAB program. To achieve a less subjective and more stable choice of the regularization parameter, Tikhonov regularization was carried out applying Bayesian theory combined with an estimation of the background variance of the deformation field as recently proposed (Huang et al., 2019).

Evaluation of strain energies was restricted to the cell area as outlined by the fluorescence signal of each individual cell. Cell mask binarization was performed employing the MATLAB-implemented ‘activecontour’ function. Before elementwise multiplying traction and deformation along *x*, *y* and *z*, the median of the respective cell free area was subtracted as a background noise reduction. This is important as the background energy accumulates in an area-dependent manner.

Internal stress calculation from the in-plane tractions was performed using the COMSOL Multiphysics software package with LiveLink for MATLAB. Based on the cell mask representing the projected cell, and by extruding the former into 3D with a height of 5 µm, a cell object was created that modeled the cell as a homogenous, linear elastic material. Assuming force balance, the formerly calculated traction forces were imposed as boundary load (with opposite sign) to the bottom plane of the cell object. A finite element mesh for the object was created using the automatically designed tetrahedral mesh with size setting ‘finer.’ Further, the boundary condition ‘rigid motion suppression’ was applied, and the material set to be nearly incompressible. Since small rotational artifacts could occur around an initial orientation-dependent point, the calculation was carried out for four different in-plane orientations (each orientation rotated by 90°) of the cell object. Omitting the artificial regions for each rotation, the final result was derived as an average of all rotations. The in-plane stress tensor at the bottom plane of the cell object was extracted from the solution of the finite element equations. The internal stress presented in the results is given as the average normal, principal stress at each point (i.e. the mean of the *xx*- and *yy*-component of the stress tensor). For faster computation times, the resolution of the traction force input data was reduced by a factor of ten (object size was accordingly reduced for calculation), and as a result, the resolution of the resulting internal stress output was naturally also reduced.

### Slope calculation

As a measure of U*z* curvature, we quantified the slope at each coordinate. To this end, we resized the U*z* map to its original 21-pixel resolution and adapted the unit to pixel length. From the gradient fields along *x* and *y*, respectively, we geometrically derived the dimensionless *xy* surface slope. Resizing back yielded a map that could be used for comparison. Due to limitations in *z* resolution, frames with an indentation depth of less than 0.1 µm were excluded for the T*xy*–U*z* slope correlation calculation.

### Actin and traction peak distance calculation

Actin punctae were detected from the Lifeact–EGFP fluorescence channel in Fiji (Schindelin et al., 2012). To this end, noise was reduced by means of a median filter with a radius of one pixel, followed by background subtraction (rolling ball radius of three pixels). Particles were detected and tracked using the ‘MosaicSuite’ particle tracking plugin (Sbalzarini and Koumoutsakos, 2005). The radius was set to five, the linked range to two, and the displacement to ten pixels. The percentiles were chosen for each image stack individually to account for differences in contrast. The result table was exported and further processed in MATLAB to only count particles that were tracked over at least six frames (i.e. 30 s) and detected in at least three of them. To account for this filter, the first and last six frames of the time series were ignored from the peak detection, and thus 188 frames were analyzed.

Local traction peaks were detected in MATLAB. First, all signal peaks were identified using ‘imregionalmax’ after dividing by a gross Gauss-filtered self. A relative threshold was set to 50% above local background, and an absolute threshold was defined as twice the median value within the cell mask. To avoid overestimates in cells with low total tractions, the threshold was required to be above 50 Pa. Of all detected traction peaks, only the largest peaks within a distance of 2 µm were considered, to account for the spatial resolution of the method.

Finally, as a measure of colocalization, we computed the nearest distance between detected actin punctae and traction peaks using a MATLAB-implemented *k*-nearest neighbor search (‘knnsearch’).

### TFM simulation and quantification of spatial resolution

3D reference bead stacks (*n*=5) were generated matching real data (11 *z* planes, voxel size 0.108×0.108×0.5 µm). To this end, 30 µm x 30 µm sized gel surfaces were designed to contain densely (200, 400 or 600 beads per 100 µm^2^) and vertically spread (σ in *z*: 0.1 µm) random points. The point intensities were heterogeneous, and after application of a Gaussian filter along *x*, *y* and *z*, followed by a normalization, mimicked our experimental images. Background noise was added to keep the simulation as accurate as possible (Fig. S1A).

Tractions were simulated as four circular foci aligning along the corners of centrally located squares (Fig. S1B). Inverse to the TFM calculation, the Green's function matrix was created to generate deformation fields (Jorge-Peñas et al., 2015) based on empirically set parameters (Young's modulus, 3 kPa; Poisson ratio, 0.49; *xy* pixel width, 0.108 µm; *z* pixel depth: 0.5 µm). The *z* traction magnitude was empirically set to 50% of the planar (*xy*) traction magnitudes. Applying the deformation fields in various possible sets of applied traction magnitudes (T*xy*: 0.1, 0.25, 0.5, 0.75 and 1 kPa), radii (0.3, 0.5, 0.9, 1.3 and 2.2 µm) and square widths (0.7, 1.1, 1.7, 2.6, 3.4, 4.3 and 6.5 µm) to the reference bead stacks yielded deformed images that were analyzed by 2.5D TFM. To determine the spatial resolution, we defined it as the proportion of signal peaks (T*xy* or U*z*) that could be resolved for each of the traction areas across the ten conditions (five bead stacks, centripetal and centrifugal orientation each) (Fig. S1D,E). Peaks were detected using MATLAB's ‘imregionalmax’ function and selected if meeting certain criteria. Resolved points were required to be located within the area of the applied tractions in order to be counted. Additionally, their assigned value had to surpass a relative as well as an absolute threshold, which were defined with respect to the background and foreground percentiles. Every value smaller than two times the 90th percentile of the background was defined as noise. Peaks that were than detected in the remaining areas had to be greater than 70% of these remaining values to be counted. Recovery of magnitude was then evaluated comparing the resolved T*xy* or U*z* magnitudes across all distances if at least five were resolved for a given traction radius (Fig. S1C). Finally, *z* resolution was further assessed by investigating the *z* distribution in cell-free areas by comparison of the 2.5th and 97.5th percentiles (Fig. S1F).

### Buckling instability

To establish whether the forces generated in the coupled system of cells to the substrate are sufficient for the proposed buckling, we used a classical energy approach comparing the energy required for the buckled situation with the energy needed in a simple 2D compression. First, we establish the energy contributions for bending the cell cortex, indenting the substrate and compressing the cell cortex. Estimating the contribution shows that the bending of the cortex is negligible compared to the indentation of the substrate. Balancing the energy contribution leads to a critical traction force at which buckling is expected.

Energy to bend the actin cortex: the bending energy of a thick layer is defined as 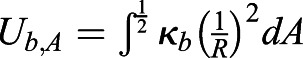, where *κ*_*b*_ is the bending modulus, *R* is the radius of curvature for the bending and *A* is the area considered. Simple geometry shows that 
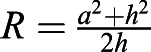
, where *a* is the radius of the cell and *h* is the indentation from the unbent situation (Fig. 5B). Assuming a constant curvature and the limit of *a*≫*h*, the integral simply yields the area of the cell, resulting in 
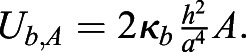


This energy is found to be small compared to substrate deformation energy, which can be derived using the Hertz solution for the force to indent an infinite half plane with a sphere: 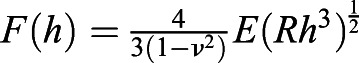, where *E*, *ν* are the Young's modulus and Poisson's ratio of the substrate, respectively. Approximating the Poisson's ratio with 0.5 and integrating the force over the indentation, keeping in mind that 
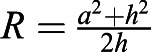
, where *a*≫*h*, yields the deformation energy as 
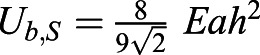
.

The energy to compress the actin cortex is simply given by *U*_*c*_=*κ*_*A*_×Δ*A*, using the compression modulus *κ*_*A*_.

Using known values of these parameters either from literature or as measured here, we see that the bending of the cell cortex is negligible compared to energy required for indenting the substrate. The explicit values used are *κ*_*b*_=*κ*_*A*_*t*^2^, where *t*=100 nm is the thickness of the actin cortex, and *κ*_*A*_=*E*_*a*_*t* can be calculated from a stiffness *E*_*a*_≈1 kPa of the actin cortex; *h*≈0.25 μm, *a*≈5 μm and *A*=*πa*^2^ describe the geometry of the cell; and *E*=3 kPa is the Young's modulus of the substrate.

The buckling will then happen at a critical traction stress. Beyond this critical stress, it is more energy efficient to buckle than to compress the actin network. This critical tension can be calculated by equating the bending and the compressing energy and keeping in mind that the area change can be calculated using the conservative approximation that the traction force is fully applied on the actin cortex *σ*=*E*_*a*_*u*, where 
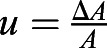
 is the strain that is approximated to the relative area change in the compression situation. Hence the buckling condition is *U*_*c*_>*U*_*b*_, which yields 
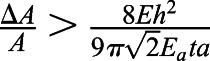
, leading to the critical traction force for the given parameters of 
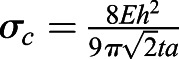
. In the given situation we would hence expect the system to buckle if the traction forces are larger than 75 Pa. It should be noted that we have used conservative estimates here, meaning that the real buckling might happen much earlier.

### Immunofluorescence and microscopy

LPS-treated Lifeact–EGFP-labeled ER-Hoxb8-derived monocytes (day 3) were seeded onto 3 kPa PAA gels (25,000 cells per gel) and incubated for 1 h. The cells were then washed once with calcium- and magnesium-free PBS (PBS^−/−^) and fixed in 4% paraformaldehyde (PFA) for 15 min at room temperature. Afterwards, the sample was washed three times with PBS^−/−^ and blocked for 1 h at room temperature using PBS^−/−^ supplemented with 20% goat serum (GS, Sigma, St Louis, USA) and 0.2% Triton-X-100 (Carl Roth, Karlsruhe, Germany). For mouse-on-mouse staining, the cells were blocked for 1 h at room temperature using an unconjugated goat anti-mouse IgG H&L antibody (#ab6708, 1:1000; Abcam, Cambridge, UK) diluted in the previously mentioned blocking solution. Next, the samples were incubated with primary antibodies against vinculin (#V9264, 1:100; Sigma, St Louis, USA) or talin-1 (#ab71333, 1:100; Abcam, Cambridge, UK) diluted in blocking solution for 1.5 h at room temperature or overnight at 4°C.

After three washes with PBS^−/−^, the samples were incubated with the appropriate secondary antibody (#A21124, 1:500 or #A11011, 1:500; Thermo Fisher Scientific, Waltham, USA) diluted in blocking solution for 45 min at room temperature. F-actin was stained using phalloidin (#ab176753, 1:1000; Abcam, Cambridge, UK). Finally, the samples were washed three times with PBS^−/−^.

Images were acquired using the Slidebook 6 software (3i, Denver, USA), and two setups with the same specification comprising an inverted microscope (Nikon Eclipse Ti-E, Minato, Japan) equipped with a CSU-W1 spinning-disk head (CSU-W1 Yokogawa, Musashino, Japan) and a scientific CMOS camera (dynamic TFM experiments: Orca-flash4.0 v2; Hamamatsu Photonics K.K., Japan; other TFM experiments and immunostaining: Prime BSI, Photometrics, Tucson, USA). A 60× Plan Apo water-immersion objective (Nikon, Minato, Japan) with a numerical aperture of 1.2 and excitation LASER of 488 nm/561 nm wavelength were employed.

Images were analyzed and prepared for publication using the open-source software Fiji.

### Statistical analysis and visualization

Independent TFM experiments were defined as experiments carried out on different days. Additionally, cells from at least three PAA gels were analyzed per condition. Significance was tested by employing the MATLAB-implemented Mann–Whitney U-test (‘ranksum’). For testing the bending versus buckling mode by COM distance ratios, a two-tailed one-sample *t*-test (MATLAB ‘ttest’) was calculated. The significance level α was set to 0.05. *P<0.05, **P<0.01, ***P<0.001 throughout the study. Boxplots were generated using the MATLAB ‘boxplot’ function. The box visualizes the 25th and 75th quantiles in addition to the median value. The whiskers extend to the data points that are closest to but are not counted as outliers (i.e. 1.5 times the interquartile range smaller than the 25th quantile or greater than the 75th quantile). Error bars reflect the mean±s.d. Pearson correlation coefficients were calculated using the MATLAB-implemented ‘corr’ function. All heatmaps were created using perceptually uniform colormaps (https://www.mathworks.com/matlabcentral/fileexchange/51986-perceptually-uniform-colormaps) to accurately visualize quantities. The data supporting our findings, and the code utilized in this study, are available from the authors upon reasonable request.

## Supplementary Material

Supplementary information

Reviewer comments
