# Supplementary material for: Analysis of monocyte cell tractions in 2.5D reveals mesoscale mechanics of podosomes during substrate-indenting cell protrusion
Source: J Cell Sci. 2022 May 27;135(10):jcs259042. doi: 10.1242/jcs.259042 (PMC9189428; doi:10.1242/jcs.259042)
Supplement: Supplementary information [file joces-135-259042-s1.pdf]

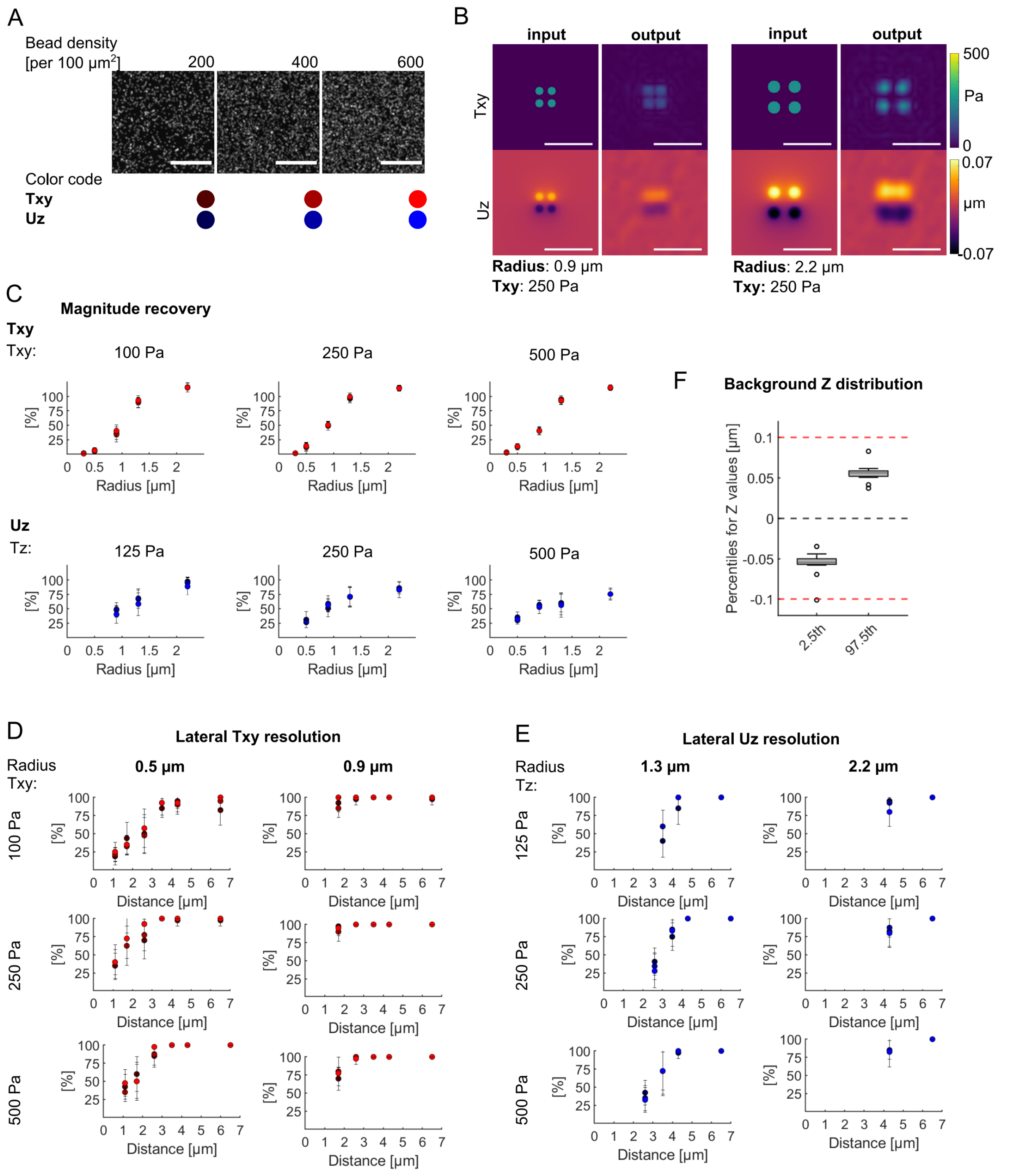

Fig. S1. TFM-simulation experiments allow for evaluation of 2.5D resolution. (A) 3D reference bead stacks were generated by empirical properties. Displayed are the reference 2D projections with bead densities ranging from 200 to 600 beads per 100 µm^2 (n = 5 simulated stacks per density) and their color code as used in the plots. Scale bar: 10 µm. (B) Example comparison of simulated four-point tractions and

recovered signal by traction radius and in-plane traction magnitude. Scale bar: 10 µm. (C) Percentage of recovered Txy or Uz magnitude across all distances by traction radius for more than 5 resolved tractions per radius. While in-plane tractions can be resolved over a broad spectrum, tractions above a 2 µm radius are overestimated. Indentation is more difficult to resolve, but indentation is not overestimated. The decrease in recovery likely arises due to predominant distortions because of the untypically strong in-plane tractions of 500 and 1000 Pa. (D) The lateral in-plane resolution for radii of 0.5 and 0.9 µm, which are similar to podosomes in size, range at around 3 µm and 2 µm, respectively. Notably, increasing traction magnitudes does not improve resolution indefinitely as seen for 500 Pa and 0.9 µm due to force field interference. (E) The lateral resolution for Uz is substantially lower and ranges at around 4 µm. However, as we are primarily interested in broad indentations, this resolution works quite well. (F) More important for this focus is what magnitude of resolved deformation can be distinguished from the background. A descriptive analysis of the z value distributions in cell-free areas reveals a 95% interval of around ± 0.05 µm with a safety zone to ± 0.1 µm (n = 12 positions from four independent experiments). Error bars: mean ± s.d.

Journal of Cell Science • Supplementary information

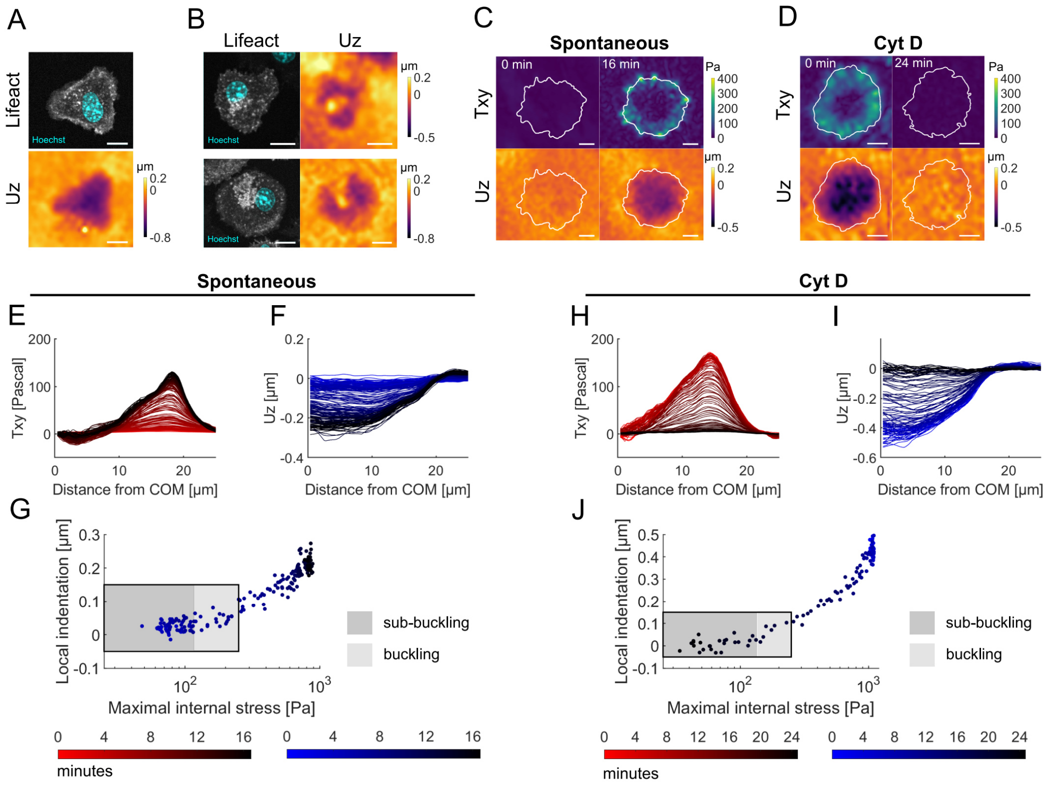

Fig. S2. Actomyosin-dependent buckling is supported by special cases. Some cells appear (A) in shapes or (B) with irregular deformation patterns that are unlikely or even impossible to be achieved by bending, and instead can be visually linked to actin organization. (C) In individual cells, spontaneous indentation or (D) CD-induced loss of indentation expose radial profiles that show peripherally located tractions (E+H) and broad indentations (F+I) throughout the time series. (G+J) Plotting the maximal internal stress per frame against its local indentation (mean of 4 µm x 4 µm) indicate a kink at around 100 Pascal (insets). This might correspond to the postulated buckling threshold of 75 Pa. Scale bars: 10 µm.

Journal of Cell Science • Supplementary information

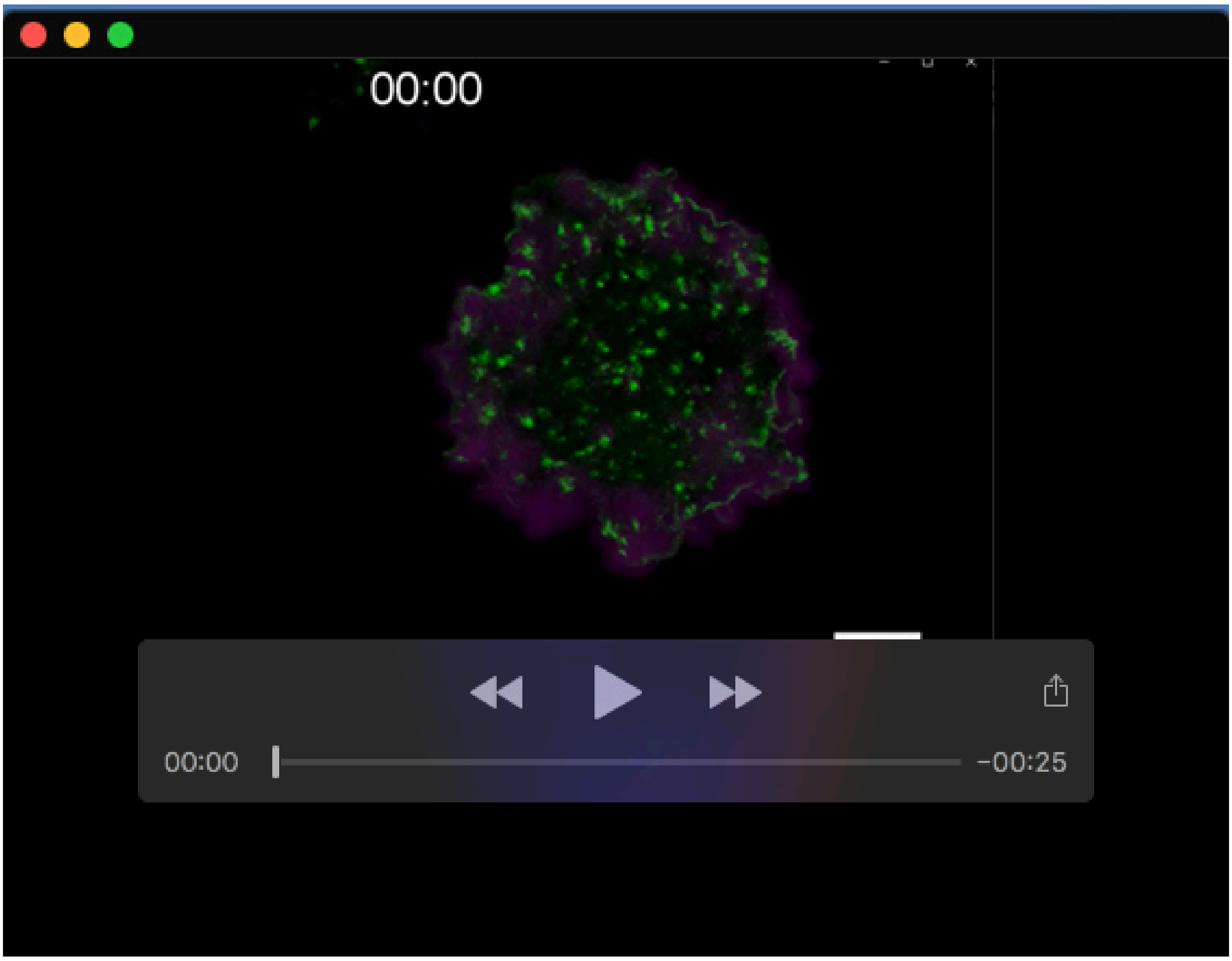

Movie 1. Dynamic podosomes colocalize with traction hot spots. Shown is an example cell from Fig. 2 with surface-projected Lifeact-EGFP (in green) and overlayed in-plane tractions (in magenta). Capturing interval: 5 seconds. Scale bar: 10 µm.

Journal of Cell Science • Supplementary information

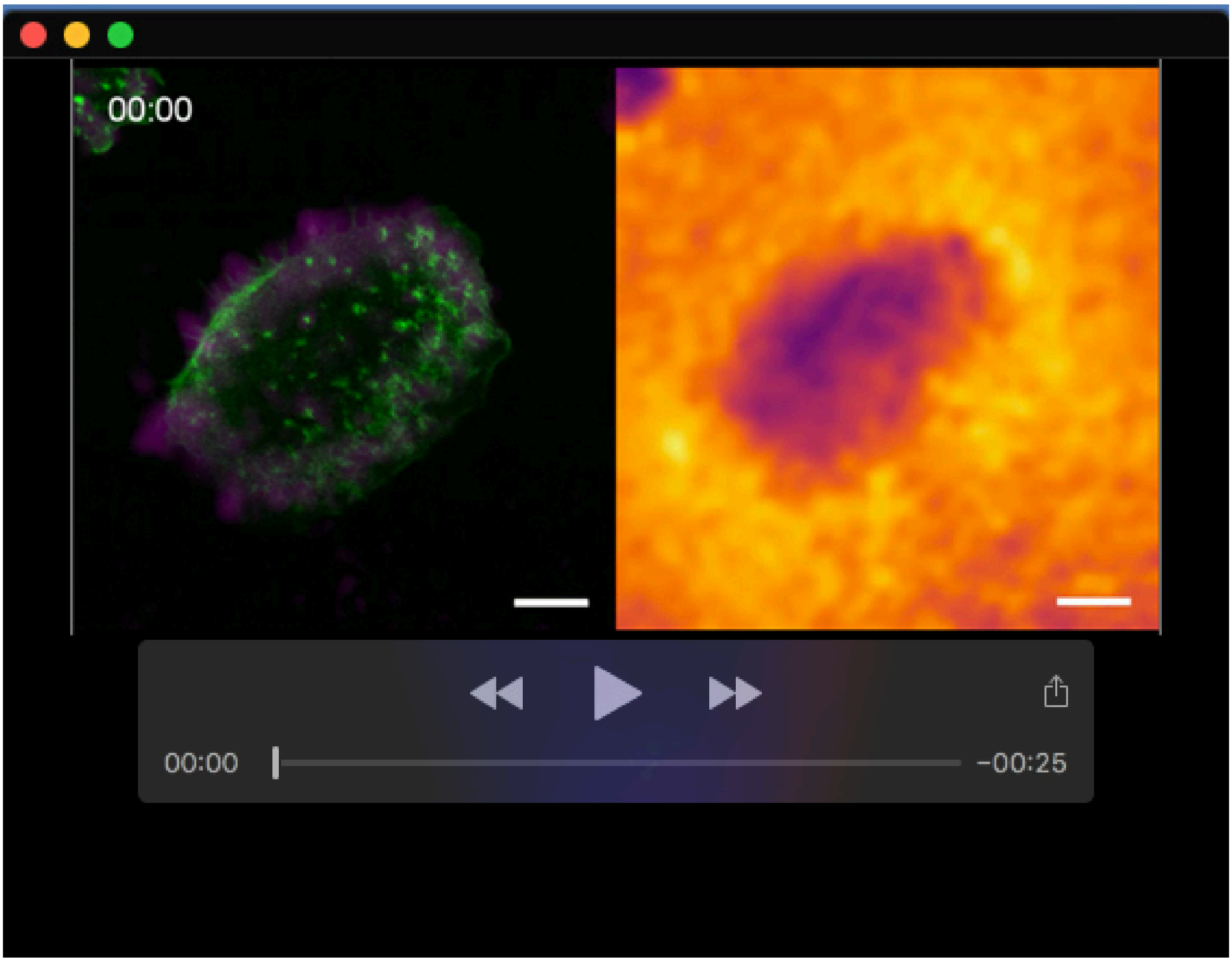

Movie 2. Relation of podosomes, in-plane tractions, and cell-wide indentation. *Left:* Shown is an example cell from Fig. 2 with surface-projected Lifeact-EGFP (in green) and overlayed in-plane tractions (in magenta). *Right*: The resolved indentation reveals a broad, heterogeneous protrusion into the substrate. Over time, small elevations from the background can be linked to actin dynamics and in-plane tractions. Capturing interval: 5 seconds. Scale bar: 10 μm.
