# Supplementary material for: Analysis of monocyte cell tractions in 2.5D reveals mesoscale mechanics of podosomes during substrate-indenting cell protrusion
Source: J Cell Sci. 2022 May 27;135(10):jcs259042. doi: 10.1242/jcs.259042 (PMC9189428; doi:10.1242/jcs.259042)
Supplement: Reviewer comments [file joces-135-259042_review_history.pdf]

Analysis of monocyte cell tractions in 2.5D reveals mesoscale mechanics of podosomes during substrate-indenting cell protrusion

Hendrik Schürmann, Fatemeh Abbasi, Antonella Russo, Arne D. Hofemeier, Matthias Brandt, Johannes Roth, Thomas Vogl and Timo Betz
DOI: 10.1242/jcs.259042

Editor: Arnoud Sonnenberg

Review timeline

Original submission:	18 June 2021
Editorial decision:	2 August 2021
First revision received:	23 February 2022
Editorial decision:	5 April 2022
Second revision received:	27 April 2022
Accepted:	27 April 2022

Original submission

First decision letter

MS ID#: JOCES/2021/259042

MS TITLE: 2.5D Tractions in monocytes reveal mesoscale mechanics of podosomes during substrate indenting cell protrusion

AUTHORS: Hendrik Schürmann, Antonella Russo, Arne D. Hofemeier, Matthias Brandt, Johannes Roth, Thomas Vogl, and Timo Betz
ARTICLE TYPE: Research Article

We have now reached a decision on the above manuscript.

To see the reviewers' reports and a copy of this decision letter, please go to: https://submit-jcs.biologists.org and click on the 'Manuscripts with Decisions' queue in the Author Area. (Corresponding author only has access to reviews.)

As you will see the reviewers consider your findings of interest but at the same time raise several substantial concerns. These include the spatial resolution that can be achieved by 2.5D TFM, (2) the role of the nucleus in the bending and buckling models, (3) the evidence that the cortical actin and the interpodosomal actin networks are separate entities, etc. etc. Finally, reviewer 3 mentions that the manuscript is hard to read and comprehend, and suggest to have it checked by a native English speaker.

Because of these concerns and additional experimentation required to support your conclusions, I am returning the manuscript to you to decide whether you wish to submit a revised version of your manuscript. I would certainly be interested in receiving a revised manuscript if you are able to address all the reviewer comments in full. I will then return it to the original reviewers for further comments.

We are aware that you may be experiencing disruption to the normal running of your lab that makes experimental revisions challenging. If it would be helpful, we encourage you to contact us to discuss your revision in greater detail. Please send us a point-by-point response indicating

© 2022. Published by The Company of Biologists under the terms of the Creative Commons Attribution License (https://creativecommons.org/licenses/by/4.0/).

where you are able to address concerns raised (either experimentally or by changes to the text) and where you will not be able to do so within the normal timeframe of a revision. We will then provide further guidance. Please also note that we are happy to extend revision timeframes as necessary.

Please ensure that you clearly highlight all changes made in the revised manuscript. Please avoid using 'Tracked changes' in Word files as these are lost in PDF conversion.

I should be grateful if you would also provide a point-by-point response detailing how you have dealt with the points raised by the reviewers in the 'Response to Reviewers' box. Please attend to all of the reviewers' comments. If you do not agree with any of their criticisms or suggestions please explain clearly why this is so.

<u>Reviewer 1</u>

Advance summary and potential significance to field

In this manuscript, Schurmann et al use 2.5D traction force microscopy to study mechanical aspects of podosomes. Using a self developed Matlab algorithm they calculate in-plan and out-of-plane forces in podosome clusters. Based on these calculations, they conclude that the traction forces are not explained by the sum of the individual podosome mechanics but rather by a superior mesoscale podosome organization. Also, they show that the in-plane and out-of-plan traction force geometry suggests a buckling induced protrusion behaviour.
Lastly, the authors propose a model for podosome mesoscale dynamics based on the presence of an interpodosomal actomyosin network.

The applied method of 2.5TFM is an interesting approach and could be usefull in the study of protrusive structures such as podosomes and invadopodia. Also the mesoscale mechanics model is interesting and may indeed explain the function of the interpodosomal actin filaments for which currently no explanation is provided in previous studies.

Comments for the author

Although the mesoscale mechanics model is interesting the data to support this model is quite preliminary, contrast previous findings and is mostly descriptive. Specifically, some conclusions seem to be derived from a single cell or only a few cells (Fig 3A, 3B?, 4A-C?, Fig 5?, Fig. 6E), which makes it really hard to assess the robustness of the conclusions. Further, the cluster wide protrusions contrasts previous findings by the Poincloux group. Although the authors claim that the single podosome protrusions are masked by the cluster wide protrusion, the cluster wide protrusion was not observed with the protrusion force microscopy and an explanation for this difference is warranted. Finally, this study would greatly benefit from some chemical perturbations, for example inhibiting adhesion or contraction to make the study less descriptive and to provide a better foundation for some of the conclusions.

Specific additional major comments:
- Introduction and abstract could be more focussed towards podosomes.
Invadopodia are also introduced but it is not clear to me why and it serves little purpose for the current study. My suggestion would be to mention invadopodia only briefly in the introduction and leave it out of the abstract.
Similar remark for the discussion, this includes quite some statements on matrix degradation while this is not studied. It is better to specifically focus on the mechanical aspects investigated in this study.
- Fig 1A is not really informative, I would suggest to leave it out.
Fig 1B (with the corresponding description in the introduction) could be moved to the results and fused with the current Fig. 2. In this way, no Figures are referenced in the introduction.
- The explanation for how the forces are calculated is hard to understand. The panels are not well organized and it is also unclear what is represented in the images at some occasions (Fig 2A, what are the planes?;

© 2022. Published by The Company of Biologists under the terms of the Creative Commons Attribution License (https://creativecommons.org/licenses/by/4.0/).

Fig. 2B, is this a schematic image or extracted from real data?; Fig. 2D, how is the stress image derived from the traction forces?;

- Although presented as an example movie, quite some conclusions are derived from Supplementary Movie 1 related to the direction and magnitude of the traction forces. Yet, this is only one movie and to derive conclusions from “ring” or “belt” mechanics more traction force measurements are required.

Further, it is unclear how the overall conclusion “Taken together, the temporal evolution of the traction force pattern and the disperse scattering of the actin signal into apparently individual podosomes suggests that a mesoscale effect is dominating over forces originating from individual podosomes.” is reached also the statement “The mesoscale mechanical effect was most apparent upon podosome cluster formation” is poorly backup up by the results.

- Overall, the images are sometimes a little hard to interpret. The traction forces in Fig. 3A are very small, same as for the green and red circles in 3B and the podosomes in 3C.
- Figure 3C, the vinculin signal and, to a lesser extent, the talin signal seems to overlap with the actin signal while actin is known to be present in the core and vinculin and talin should be in the ring.
- It would be informative to add some actin/vinculin/talin pictures of S100A8/A9 stimulated cells as well and quantify some basic parameters for the two conditions such as cluster size, podosome number etc…
- Fig. 5, how do the authors explain the increase in traction forces? Is this a cell that is spreading? Also, do these hotspots of traction forces correlate with adhesion hotspots? Also, if I understand the cluster-wide protrusion correctly, shouldn’t that result in a more uniform distribution of the Txy instead of these hotspots?

<u>Reviewer 2</u>

Advance summary and potential significance to field

This manuscript by Betz and co-workers describes investigations into the mechanical activity of podosomes using 2.5D traction force microscopy. To achieve optimal resolution in z-direction, the authors performed careful fits of the intensity distribution along confocal z-stack. The recorded displacement fields were then converted into traction stress using established algorithms. Traction fields were compared and correlated to expression patters of relevant cell constituents (actin vinculin, talin). The stress patterns and their temporal dynamics are analysed in detail.

Comments for the author

The present manuscript adds useful information to the known literature, but I do not think it can be published in its current form. My main concern is that the authors do not quantify the in-plane stress resolution of their 2.5D TFM modality, and that from what is known about TFM in the literature, it is likely that their measurement has insufficient spatial resolution to resolve the mechanical force exerted by the investigated podosomes. With this limitation, it is very difficult to draw any conclusions on whether podosomes exert force collectively or individually. I believe it is quite likely that due to the limited spatial resolution of 2.5D TFM, the force exertion points from podosomes are simply under-sampled, which would smear out the observed stress pattern. The bias against individual podosome force is exacerbated by the fact that the authors later perform rotational averaging of the applied stress around the cell’s centre of mass. When this is done, obviously any signature of individual podosome force will be smoothed out.

My concern is also based on having seen several recent literature reports on the forces / stresses exerted by individual podosomes and by (structurally very similar) invadopodia [Labernadie, A. et al. (2014), Bouissou, A. et al. (2017), Kronenberg, N.M. et al. (2017), Dalaka, E. et al. (2020), Meek, A.T. et al. (2021)]. While these reports have been able to resolve stress in vertical direction with a high in-plane resolution, stress in horizontal direction was inferred indirectly in these cases. It is therefore obviously useful and interesting to perform 2.5D TFM in order to map vertical and horizontal stress simultaneously. However, I find that the limitations of the present study need to be discussed more openly and in more detail. At present we get a short section on the “caveats” at the very end of the manuscript. However, the key findings and main conclusions listed in the abstract, the introduction and throughout the results and discussion sections, are almost all strongly impacted by these caveats. Therefore, the caveats need to be discussed much more

© 2022. Published by The Company of Biologists under the terms of the Creative Commons Attribution License (https://creativecommons.org/licenses/by/4.0/).

prominently and in conjunction with the main claims to ensure the community does not draw the wrong conclusions.
The “buckling” stress features observed in the present work resemble patterns previously also described for cells forming focal adhesion contacts [Legant, W. R. et al. (2013), Kronenberg, N. M. et al. (2017)]. This raises the question whether the observations are novel and/or specific to podosome mechanics. The authors state that the cells they study do not form focal adhesion contacts. (“However, monocytic cells as fast migrating immune cells have not been reported to exhibit stable and long-lasting stress fibers or classical focal adhesions.”, p. 19). It would be helpful to have some confirmation for this claim, including stains, a control experiment in a cell where podosome formation is suppressed (to see the absence of focal adhesion mediated stresses) and literature references showing that focal adhesions are completely absent in monocytic cells (even when the monocytes are not in a confined environment but on a flat substrate).
Related to this, in several cases the horizontal stress occurs in areas of the cell that do not show podosomes; see e.g. Fig. 7C where podosomes are located towards the cell centre (in the area with large Uz) but horizontal stress (Fxy) is at the periphery and outside the podosome-rich area. If the cell forms no focal adhesion contact points in this podosome-free region, it is difficult to envision how the cell can apply force to the substrate here as the region would then be free of any anchor points of any type.
In Fig. 6, the authors discuss a “bending model” and a “buckling model”. It is concluded that the buckling model is more probable and that the buckling occurs due to a contractile network on the ventral side of the cell. It remains somewhat unclear to which extent this network is tied to the presence of podosomes. In my view, the observed stress pattern is not specific to monocytes or podosomes, but similar patterns are in fact also observed for other cells and for focal adhesion contacts.

Minor points:
Fig. 2C shows a histogram of observed deformation in Z and claims to highlight the lower and upper limit of the 95% interval. However, these limits are themselves shown as intervals (grey areas). It remains unclear why these are regions rather than lines.
Fig. 2D shows displacement fields (Ux, Uy, Uz) and the traction stress obtained from these (Txy and Tz). The panel also shows a further illustration of “stress”.
It remains unclear what is meant by this and how “stress” would differ from Txy and Tz. Please explain/clarify.
Fig. 3A claims that the “captured time interval is 5s”, although the scale in the plot shows a 16min x-axis. Clarify and correct if necessary. While the time lapse images in Fig. 3A are described in the caption, nothing is said about the plot at the bottom of Fig. 3A. Please expand the caption.
Fig. 3B lacks information on the number of observations/samples/podosomes used to generate the boxplots. In addition, it would be clearer to show the image first followed by the boxplot, rather than the other way around.
The caption to Fig. 4A seems poorly written (“Though without correlation traction peaks…”).
In contrast to earlier figures, Fig. 4B discusses Uz (vertical displacement)
rather than Tz (vertical stress). Wouldn’t it be more relevant to the mechanical analysis to discuss vertical stress (and to consistently analyse one of both throughout the manuscript)?

<u>Reviewer 3</u>

Advance summary and potential significance to field

Using lifeact expressing HoxB8 derived monocytes and 2,5D force microscopy, the authors address an interesting aspect of podosome functionality. They find that the indentation a cell imposes on the substrate is not explained by single podosome pushing forces but rather by a collectively generated mechanism that seems to result from the traction forces between podosomes. Two principle models could explain this effect: the nucleus being pushed into the substrate (bending model) or buckling forces arising from interpodosomal contractile forces (buckling model). Including a simple mechanical model, the authors argue for buckling.
This seems to be thorough work and the result of deep mechanobiological considerations. The question is also interesting and if true, this is certainly an interesting aspect of podosome biology.

© 2022. Published by The Company of Biologists under the terms of the Creative Commons Attribution License (https://creativecommons.org/licenses/by/4.0/).

Comments for the author

Major points:
I have a major problem with this manuscript as I find the writing largely incomprehensible. Passages read like a word by word translation of german into english and the whole terminology is way more complicated than necessary. I spent quite some time with the manuscript and only understood fractions. I doubt that a less motivated reader will make it very far. Presumably most cell biologists will stop reading with the last sentence of the abstract, which is totally cryptic and therefore representative for the rest of the text. I personally think the text would benefit a lot from shortening 50% and simplifying language with the help of a native speaker.

I also have a problem with the very indirect way of distinguishing between the bending and buckling model.
The key here seems to be the nucleus and I do not see why this could not be directly addressed with experimentation. The authors could analyse enucleated cells or ventral areas away from the nucleus. If indentation happens without the nucleus, this would strengthen the buckling model enormously. Analysing nuclear shape in relation to the indentation could also be informative.
Another problem I had was the distinction the authors make between cortical actin and the interpodosomal actin network. In my eyes these are either tightly interlinked structures, or possible even the same structure.
The authors argue that these are two separate networks and I cannot see the evidence for this.
Another set of experiments that could be very informative is: imaging the actin network together with force microscopy and then pharmacologically disassembling actin and myosin. The spatio-temporal correlation between a gradually disassembling acto-myosin system and the relaxation of the substrate could support either one or the other model.

First revision

Author response to reviewers' comments

Reviewer #1

Advance Summary and Potential Significance to Field:

"In this manuscript, Schurmann et al use 2.5D traction force microscopy to study mechanical aspects of podosomes. Using a self developed Matlab algorithm they calculate in-plan and out-of-plane forces in podosome clusters. Based on these calculations, they conclude that the traction forces are not explained by the sum of the individual podosome mechanics but rather by a superior mesoscale podosome organization. Also, they show that the in-plane and out-of-plan traction force geometry suggests a buckling induced protrusion behaviour. Lastly, the authors propose a model for podosome mesoscale dynamics based on the presence of an interpodosomal actomyosin network.
The applied method of 2.5TFM is an interesting approach and could be usefull in the study of protrusive structures such as podosomes and invadopodia. Also, the mesoscale mechanics model is interesting and may indeed explain the function of the interpodosomal actin filaments for which currently no explanation is provided in previous studies."

Answer: We would like to thank the reviewer for the overall positive and encouraging opinion of our manuscript.

Comments for the Author:

Comment 1:
"Although the mesoscale mechanics model is interesting the data to support this model is quite preliminary, contrast previous findings and is mostly descriptive. Specifically, some conclusions seem to be derived from a single cell or only a few cells (Fig 3A, 3B?, 4A-C?, Fig 5?, Fig. 6E), which makes it really hard to assess the robustness of the conclusions."

© 2022. Published by The Company of Biologists under the terms of the Creative Commons Attribution License (https://creativecommons.org/licenses/by/4.0/).

Answer: We thank Reviewer 1 for pointing out the concerns regarding robustness of the shown results. The reviewer is certainly right that the conversion of phenotypes (former Fig. 3A+B) as mentioned in the text was unique to this one cell. This particular cell was simply used to conveniently illustrate the different phenotypes in a single cell. Hence, no final conclusions were drawn from this data. To improve the paper, we simply removed the part that introduces the complexity of the system and now rather focus on the main scope of the paper, which are podosome clusters. Throughout the revised manuscript, we now better visualize our analysis pipeline and support images by several example cells (former Fig. 4A-C, now Fig. 2D-H; former Fig. 5, now Fig. 3) and provide clear quantification with a collection of cells where necessary. To give further information about the robustness, we now explicitly point out the sample counts to better support the statistical weight of the conclusions. In particular, the special case of former Fig. 6E was complemented by a reverse event and moved to Fig. S2 to only include robust results in the main part.
We furthermore agree that at first sight, some results seem to contrast previous findings. However, as we explain below, in many of these cases the reason for the contrasts can be found in the different viewpoints and research questions leading to details in the data analysis that resulted in such impressions. Furthermore, the now included actomyosin perturbation experiment as well as the nuclear analysis should make the study less descriptive and lead to improved robustness of the results.

Comment 2:
"Further, the cluster wide protrusions contrasts previous findings by the Poincloux group. Although the authors claim that the single podosome protrusions are masked by the cluster wide protrusion, the cluster wide protrusion was not observed with the protrusion force microscopy and an explanation for this difference is warranted."

Answer: Reviewer 1 points out the apparent inconsistency of our protrusion findings with current literature and, in particular, *protrusion force microscopy* (Labernadie et al., 2014; Proag et al., 2015), but also *ERISM* (Kronenberg et al., 2017). However, as we now explain in more detail in the manuscript, instead of contradicting, our study in fact complements previous findings. Looking at the details of the experiments and the analysis of these previous reports are helpful to better illustrate this point.
While *Kronenberg et al.* described, fully consistent with our results, "an unstructured broad pushing of the main cell body into the substrate and pulling at the cell periphery" in podosome-forming macrophages, the aim of their paper was to focus on single podosomes and the method's high resolution. Hence, they applied a spatial Fourier filter with a cut-off frequency of 0.5 μm^{-1} to segment out the single podosome contributions which they were able to resolve. Therefore, all information regarding cluster-wide indentations were neglected to better study the properties of single podosomes. Similarly, previous *protrusion force microscopy* studies as carried out by the Poincloux group focused on single podosomes. While they did not describe the broad indentation, this work also removes cell scale deformation by subtracting a third- order polynomial from the height/z map for each line of measurement to level the images. Hence, as the focus of these investigations was on the level of single podosomes, the data treatment was optimized to best study them. Although a broad indentation as found in our study is not described in these papers, the research question and the analysis were elegantly adapted to remove any such signs from these studies at the outset.
Hence, our work now complements these important previous results by investigating forces on the full cell scale without denying the correctness of these previous findings. We simply extend these previous results. To better explain this, we now discuss these differences in the revised version.

Comment 3:
"Finally, this study would greatly benefit from some chemical perturbations, for example inhibiting adhesion or contraction to make the study less descriptive and to provide a better foundation for some of the conclusions."

Answer: We thank the reviewer for this constructive criticism, and we performed a series of new experiments where we perturbed the actomyosin network in different ways. In detail, we now disrupted the actin cytoskeleton using cytochalasin D (2 μM) and block contractility via Y27632 and ML7 (20 μM, and 10 μM) similar to previous studies (Labernadie et al., 2014; Meddens et al.,

© 2022. Published by The Company of Biologists under the terms of the Creative Commons Attribution License (https://creativecommons.org/licenses/by/4.0/).

2016). In the newly added figures, we observed after drug treatment a substantial decrease and even loss of indentation (Fig. 4F+H) accompanied by a decrease in strain energy by more than one order of magnitude (Fig. 4I+J). This supports the suggested role of actomyosin for in-plane traction and out-of-plane deformation generation.

“Specific additional major comments:”
Comment 4:
“Introduction and abstract could be more focussed towards podosomes. Invadopodia are also introduced but it is not clear to me why and it serves little purpose for the current study. My suggestion would be to mention invadopodia only briefly in the introduction and leave it out of the abstract.”

Answer: We are grateful for this advice and changed it accordingly.

Comment 5:
“Similar remark for the discussion, this includes quite some statements on matrix degradation while this is not studied. It is better to specifically focus on the mechanical aspects investigated in this study.”

Answer: We thank the reviewer for this advice and now only refer to the implication that our model might have on degradation with respect to previous findings.

Comment 6:
“Fig 1A is not really informative, I would suggest to leave it out. Fig 1B (with the corresponding description in the introduction) could be moved to the results and fused with the current Fig. 2. In this way, no Figures are referenced in the introduction.”

Answer: In agreement, we changed our figures according to this advice in Fig. 1 (former Figs 1+2).

Comment 7:
“The explanation for how the forces are calculated is hard to understand. The panels are not well organized and it is also unclear what is represented in the images at some occasions (Fig 2A, what are the planes?;”

Answer: We agree that the method is an important part of the manuscript that needs to be clearly presented. We revised the respective figures and text with the aim of a better, easy-to-follow illustration (Fig. 1) and explanation, where we more refer to the literature where possible to better point out the novelty we developed throughout this project. The mentioned planes (former Fig. 2A, now Fig. 1B) refer to neighbored image planes of the z stack and should emphasize the visibility of substrate indentation. We changed it to ‘Z stack planes’ instead, and we aligned it vertically for a more intuitive visualization. Additionally, we now focus more strongly on the workflow itself and traction as well as indentation calculation. The z resolution part was moved to Fig. S1 along with the new section about the xy resolution. While it is not essential for understanding of the findings, it provides deeper insights into the employed method’s capabilities.

Comment 8:
“Fig. 2B, is this a schematic image or extracted from real data?; ”

Answer: The top image in Fig. 1C (former Fig. 2B) is indeed a schematic presentation, which we now indicate by a label. Real data calculated from the cell shown in Fig. 1B are shown in Fig. 1C IV-VI (former Fig. 1D).

Comment 9:
“Fig. 2D, how is the stress image derived from the traction forces?; ”

Answer: The internal (normal) stress displayed in Fig. 1C VI (former Fig. 2D) is computed from the in-plane traction stress (Txy) by use of a finite-element method (FEM) as described in previous studies (Tambe et al., 2013). To better point this out, we now state this more clearly in the text and the figure. This established method allows for estimating the stress transmission

© 2022. Published by The Company of Biologists under the terms of the Creative Commons Attribution License (https://creativecommons.org/licenses/by/4.0/).

within the cell. Briefly, conservation of momentum (Newton's law) requires that the traction forces that are applied by the cell on the substrate need to pass through the cell, which can be calculated assuming a homogeneous material inside the cell.

Comment 10:
"*Although presented as an example movie, quite some conclusions are derived from Supplementary Movie 1 related to the direction and magnitude of the traction forces. Yet, this is only one movie and to derive conclusions from "ring" or "belt" mechanics more traction force measurements are required. Further, it is unclear how the overall conclusion "Taken together, the temporal evolution of the traction force pattern and the disperse scattering of the actin signal into apparently individual podosomes suggests that a mesoscale effect is dominating over forces originating from individual podosomes." is reached also the statement "The mesoscale mechanical effect was most apparent upon podosome cluster formation" is poorly backup up by the results.*"

Answer: We kindly thank Reviewer 1 for this comment. Indeed, the first movie which was extensively analyzed in former Fig. 3A+B represented a single and even rare example. However, we did not intend to invoke the impression that we drew substantial conclusions from this single cell. Instead, we thought of this cell and its analysis to be of instructive value and a potentially interesting example for the community on force dynamics of the respective superstructures. The conclusion we drew was based on the correlation coefficients between the Lifeact-EGFP signal, planar tractions, and the indentation (Uz) to provide a reason for investigating podosome clusters. However, as it apparently not only failed to support but even distracted from our findings, we removed the example from the manuscript. As a result, former Figs 3+4 were compressed and adapted into the revised Fig. 2.

Comment 11:
"*Overall, the images are sometimes a little hard to interpret. The traction forces in Fig. 3A are very small, same as for the green and red circles in 3B and the podosomes in 3C.*"

Answer: We adjusted the contrasts and choice of colors for Fig. 2, which contains the supportive graphics from former Fig. 3.

Comment 12:
"*Figure 3C, the vinculin signal and, to a lesser extent, the talin signal seems to overlap with the actin signal while actin is known to be present in the core and vinculin and talin should be in the ring.*"

Answer: Reviewer 1 raises valid concerns about the podosome staining. We were also surprised at first that we did not observe a clear core/ring pattern, but we attribute this to the imaging modalities on the soft PAA gels and the 3D images taken. Briefly, the small unevenness in the gel in combination with the stage skew together with potential minor (e.g., osmotic) distortions of the gel due to the PFA fixation result in the podosomes not being captured orthogonally, but possibly tilted for all resolved signals. We now include new images on soft gels that are slightly better (Fig. 2A+B), but we also emphasize the occurrence of colocalization and ring formation. We now discuss this in the text. Additionally, we repeated the fixation on cells cultured on glass (Rev. Fig. 1, below), where we indeed find results that are consistent with literature, further suggesting that these differences are due to the softness of the gels.

© 2022. Published by The Company of Biologists under the terms of the Creative Commons Attribution License (https://creativecommons.org/licenses/by/4.0/).

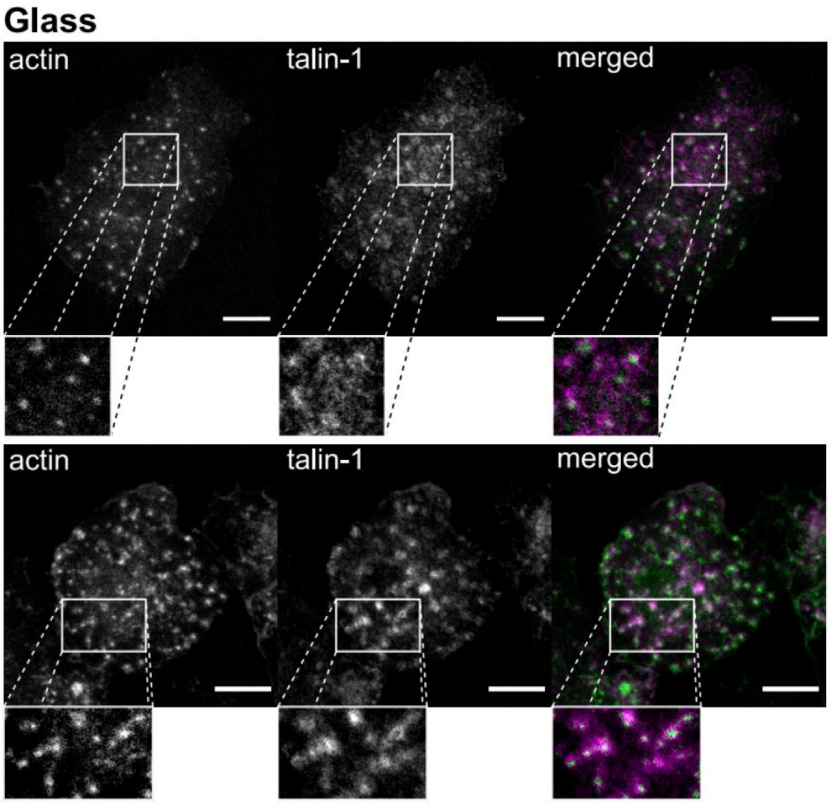

Rev. Fig. 1 LPS-treated ER-Hoxb8 monocytes seeded on glass display talin-1 and vinculin rings. Cells were fixed with PFA and stained for actin (by fluorescent phalloidin) as well as talin-1 (*top*) or vinculin (*bottom*), respectively. Scale bar: 10 µm.

Comment 13:
"It would be informative to add some actin/vinculin/talin pictures of S100A8/A9stimulated cells as well and quantify some basic parameters for the two conditions such as cluster size, podosome number etc…"

Answer: Indeed, the S100A8/A9 condition deserves a more thorough investigation. Unfortunately, from the feedback it appeared to make the story unnecessarily more complicated and to raise more questions than provide answers. Hence, we decided on leaving out S100A8/A9 from this study and fully focusing on the mesoscale mechanical model.

Comment 14:
"Fig. 5, how do the authors explain the increase in traction forces? Is this a cell that is spreading?"

Answer: As we measured the cells soon after seeding, spreading might indeed explain traction dynamics to a certain extent, as suggested by Reviewer 1. Moreover, cells naturally vary in traction magnitudes over time (Rev. Fig. 2, *Cell 1*). Tractions can even oscillate in magnitude, as has been shown for single podosome protrusion (Labernadie et al., 2014). Such oscillations can also be observed in some of our cells on a cell-wide scale (Rev. Fig. 2, *Cell 2*). The force dynamics were suggested to be attributable to mechanosensing, as they modulate the loading rate at cell adhesions (Andreu et al., 2021).

© 2022. Published by The Company of Biologists under the terms of the Creative Commons Attribution License (https://creativecommons.org/licenses/by/4.0/).

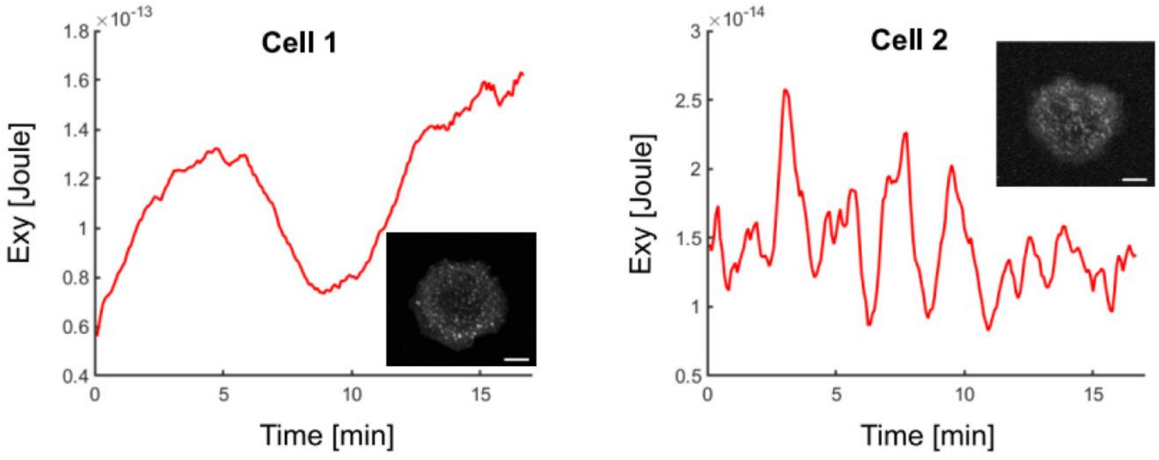

Rev. Fig. 2 LPS-treated ER-Hoxb8 monocytes seeded on PAA gels display dynamical traction magnitudes. The in-plane strain energies (*Exy*) of two example cells are plotted over time. In addition to slow dynamics (*Cell 1*), oscillations with periods of ~2 minutes can also be observed (*Cell 2*). The insets correspond to a time projection of the respective cells' Lifeact signal. Capturing interval: 5 seconds. Scale bar (inset): 10 µm.

Comment 15:
"*Also, do these hotspots of traction forces correlate with adhesion hotspots?*"

Answer: Briefly, traction forces mainly colocalize with actin punctae with regards to our xy resolution (Fig. 2E+F). However, not all actin punctae lead to a traction force. Based on the performed immunostaining (Fig. 2A+B), we consider the punctae as podosomes, and, therefore, putative adhesion spots. The asymmetry between traction and adhesion hotspots is not captured by a classical mathematical correlation analysis that is symmetric in nature. Instead, they display the mentioned one-sided relation as can be deduced from the mean nearest-neighbor distances compared between traction-podosome (T2P) and podosome-traction (P2T) peaks, respectively (Fig. 2F).

Comment 16:
"*Also, if I understand the cluster-wide protrusion correctly, shouldn't that result in a more uniform distribution of the Txy instead of these hotspots?*"

Answer: We understand Reviewer 1's remark as asking why there can be a discrete planar traction pattern (Txy) in the presence of a uniform protrusion pattern (Uz). In our proposed model, adhesion spots at the periphery still relay tractions (Txy) individually as anchor points of the network (Fig. 5A). In contrast, the uniform mesoscale protrusion is supposed to reflect the indentation due to the network's internally relayed stress, which is distributed more homogeneously (Fig. 3A) and does not require adhesion spots as the forces are perpendicular to the substrate. Therefore, discrete xy-traction hotspots and a homogeneous protrusion can be explained by our model. Notably, while it allows 2.5D measurements, our method intrinsically applies a low-pass frequency filter by pooling local bead intensities (Fig. 1C). This makes the indentation map (Uz) more homogeneous than it already is, as the putatively superimposed podosome protrusions are filtered out. However, the appearance of the traction force hotspots also demonstrate that we are still capable of resolving smaller details.

Reviewer #2

Advance Summary and Potential Significance to Field:

"This manuscript by Betz and co-workers describes investigations into the mechanical activity of podosomes using 2.5D traction force microscopy. To achieve optimal resolution in z-direction, the authors performed careful fits of the intensity distribution along confocal z-stack. The recorded displacement fields were then converted into traction stress using established algorithms.

© 2022. Published by The Company of Biologists under the terms of the Creative Commons Attribution License (https://creativecommons.org/licenses/by/4.0/).

Traction fields were compared and correlated to expression patters of relevant cell constituents (actin, vinculin, talin). The stress patterns and their temporal dynamics are analysed in detail."

Comments for the Author:

Comment 1:
"The present manuscript adds useful information to the known literature, but I do not think it can be published in its current form. My main concern is that the authors do not quantify the in-plane stress resolution of their 2.5D TFM modality, and that from what is known about TFM in the literature, it is likely that their measurement has insufficient spatial resolution to resolve the mechanical force exerted by the investigated podosomes."

Answer: We thank Reviewer 2 for raising these relevant concerns. We now quantified the force resolution and include this into the revised manuscript (Fig. S1). The lateral (xy) resolution of Txy as well as both the xy and the vertical (z) resolution of Uz are most important. The position resolution of the z indentation is high (0.1 µm); however, as already mentioned by the reviewer, the lateral resolution is much lower. We now quantify the lateral resolution by simulating the deformation of variable traction force sources, and then ask how far away force centers have to be for them to still be separated in our analysis. For example, circular force sources similar to podosomes had radii of 0.9 µm. In these cases, our 2.5D TFM displayed a minimal resolution of 2 µm (Fig. S1D), which is comparable to literature (Sabass et al., 2008). Further, even for radii of 0.5 µm, tractions can still be recognized and resolved in around 50% of the cases at 2 µm (Fig. S1C). The xy resolution of z is lower and ranges at around 4 µm, as expected (Fig. S1E).
This vertical resolution is substantially lower than that of methods like *protrusion force microscopy* or ERISM. However, our method is sufficient to resolve indentation on the cellular length scale, i.e., 10 µm and above. On this scale, our method not only provides an accurate z resolution for protrusion experiments, but it also offers additional information on planar tractions at a xy resolution not too far apart from the estimated lateral resolution of 1.6 µm for ERISM (Kronenberg et al., 2017).

Comment 2:
"With this limitation, it is very difficult to draw any conclusions on whether podosomes exert force collectively or individually. I believe it is quite likely that due to the limited spatial resolution of 2.5D TFM, the force exertion points from podosomes are simply under-sampled, which would smear out the observed stress pattern."

Answer: Reviewer 2 raises understandable concerns about our method's limitation and its implication on drawing conclusions. However, if the indentation would simply originate from the mentioned smearing effect, then it would depend on the density of podosomes. As we often find either a homogeneous distribution of podosomes or more podosomes at the periphery, this effect cannot explain the high indentation in the central region.
Additionally, our method can resolve local in-plane traction events at the individual podosome level (Fig. 2E) above background noise. This suggests that for the podosome density given in the measured cells, we are still in a region where we can resolve many podosomes that display a sufficiently large distance between each other. As we can robustly detect these discrete foci in the absence of similar artificial background peaks in cell-free areas, we rule out Reviewer 2's concern that a smearing effect due to undersampling plays a substantial role. In the revised manuscript, we more openly discussed the method's capabilities and limitations.

Comment 3:
"The bias against individual podosome force is exacerbated by the fact that the authors later perform rotational averaging of the applied stress around the cell's centre of mass. When this is done, obviously any signature of individual podosome force will be smoothed out."

Answer: Reviewer 2 is right with the statement that the rotational projection smooths out local events. Hence, using the projected averages for statements about individual podosomes is not possible. However, this averaging is the circular projection's purpose to allow for a cluster-wide description and visualization of rotational symmetry and the parameters that we need for our mathematical model (radius *a*, central indentation *h*) (Fig. 2H+5B). We would like to emphasize that we do not want to state that single podosomes do not push; however, here we focus on the

© 2022. Published by The Company of Biologists under the terms of the Creative Commons Attribution License (https://creativecommons.org/licenses/by/4.0/).

combined effect that can lead to an additional effect in form of the cell-wide indentation, which happens additionally to the previously described single podosome pushing.

Comment 4:
"My concern is also based on having seen several recent literature reports on the forces / stresses exerted by individual podosomes and by (structurally very similar) invadopodia [Labernadie, A. et al. (2014), Bouissou, A. et al. (2017), Kronenberg, N.M. et al. (2017), Dalaka, E. et al. (2020), Meek, A.T. et al. (2021)]. While these reports have been able to resolve stress in vertical direction with a high in-plane resolution, stress in horizontal direction was inferred indirectly in these cases. It is therefore obviously useful and interesting to perform 2.5D TFM in order to map vertical and horizontal stress simultaneously. However, I find that the limitations of the present study need to be discussed more openly and in more detail. At present we get a short section on the "caveats" at the very end of the manuscript. However, the key findings and main conclusions listed in the abstract, the introduction and throughout the results and discussion sections, are almost all strongly impacted by these caveats. Therefore, the caveats need to be discussed much more prominently and in conjunction with the main claims to ensure the community does not draw the wrong conclusions."

Answer: We kindly thank Reviewer 2 for stating this concern. Our manuscript indeed harbored plenty of space for misinterpretation. In hindsight, focusing on the caveat at the very end is not the best choice. In the revised manuscript, we now include the differences to the literature, and in particular we highlight the main focus of our study on the cell scale indentation at various locations.

Comment 5:
"The "buckling" stress features observed in the present work resemble patterns previously also described for cells forming focal adhesion contacts [Legant, W. R. et al. (2013), Kronenberg, N. M. et al. (2017)]. This raises the question whether the observations are novel and/or specific to podosome mechanics."

Answer: Reviewer 2 raises a crucial point questioning the interpretation of the observed mechanical patterns. Indeed, the mechanical patterns are derived from well-established methods and share many similarities with other cells. However, what we use here to analyze the underlying indentation mechanism (i.e., bending or buckling) is a combination of various mechanical parameters (tractions, out-of-plane deformation, internal stress) and their detailed relations. With regards to the two mentioned studies, differences can be found in the indentation patterns. Specifically, the 2.5D TFM study (Legant et al., 2013) revealed minimal central indentation, unlike our study, but substantial rotational moments at the peripheral tractions. In the ERISM study (Kronenberg et al., 2017), central indentation was observed for 3T3 fibroblasts (Kronenberg et al. 2017, Fig. 5A), but not only did the indentation colocalize with the nucleus, but the shown macrophage's indentation pattern differed considerably (Kronenberg et al. 2017, Fig. 3A). Both phenomena, the peripheral rotational moments and the sub-nuclear indentation in principle fit to the bending mechanism (Fig. 4A). This geometric relation is illustrated by computing the indentation slope (Uz slope) of a podocyte from a previous ERISM study (Haley et al., 2018): The weak yellow ring likely corresponds to the rotational moment, but the biggest indentation slope aligns around the nuclear region (Rev. Fig. 3).

***Note:** Figure provided for reviewer has been removed. The figure had been adapted from Fig. 3a, 3b and 3c from Haley, Kathryn E et al. (2018) "Podocyte injury elicits loss and recovery of cellular forces." Science advances vol. 4,6 eaap8030. 27 Jun. 2018, doi:10.1126/sciadv.aap8030*

Comment 6:
"The authors state that the cells they study do not form focal adhesion contacts. ("However, monocytic cells as fast migrating immune cells have not been reported to exhibit stable and long-lasting stress fibers or classical focal adhesions.", p. 19). It would be helpful to have some confirmation for this claim, including stains, a control experiment in a cell where podosome formation is suppressed (to see the absence of focal adhesion mediated stresses) and literature

© 2022. Published by The Company of Biologists under the terms of the Creative Commons Attribution License (https://creativecommons.org/licenses/by/4.0/).

references showing that focal adhesions are completely absent in monocytic cells (even when the monocytes are not in a confined environment but on a flat substrate).”

Answer: Reviewer 2 points out the misleading generalization of our statement. We did not intend to make a general statement about stress fibers or focal adhesions separately but their combined role in force generation. We thus weakened the wording in the revised manuscript and now refer to stress fibers in combination with focal adhesions. Additionally, we provide confirmation for this statement as asked for: (*Literature*) Stress fibers and focal contacts in combination represent the classical force transmission apparatus (Balaban et al., 2001). While for a long time it was accepted that myeloid cells do not form stress fibers at all (Blakney et al., 2012; Jones et al., 1998), it was also reported that monocyte-derived macrophages are able to form stress fibers on stiff substrates (Adlerz et al., 2015). Even from this perspective, on soft substrates like those we are using in this study (3 kPa) to our knowledge, stress fibers were not shown to play a role. (*Staining*) In a recent study (Lehtimäki et al., 2021), Lifeact-tagged cells were employed to investigate stress fiber organization. Using Lifeact-EGFP tagging, we stain for stress fibers as requested. Neither did we observe them in any of our cells nor have the phalloidin stainings exposed stress fibers.
(Further experiments) As a complete drug-induced abolishment of podosomes would certainly affect the entire actin network together with stress fibers, investigation of the LPS-untreated control appeared most reasonable to us. LPS-untreated cells (control), that form fewer or no podosomes (Rev. Fig. 4A) presented as substantially weaker compared to podosome-exposing LPS-treated cells by an order of magnitude in strain energy (Rev. Fig. 4B).

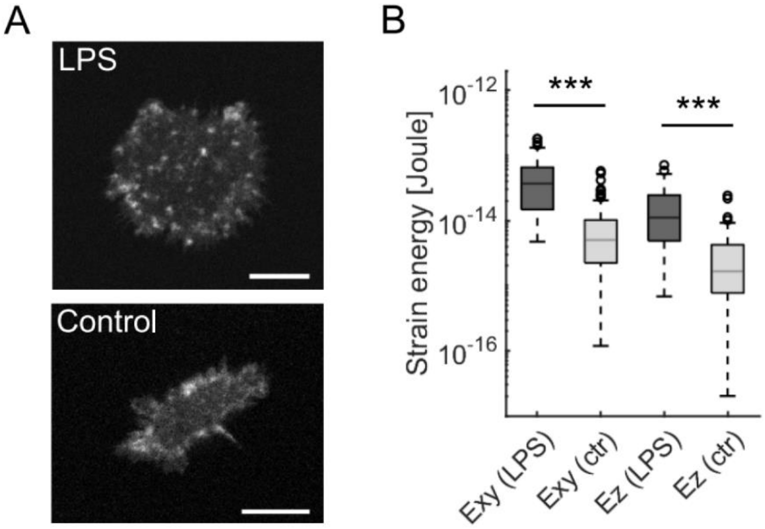

Rev. Fig. 4 LPS-treated podosome-rich ER-Hoxb8 exert significantly stronger forces than untreated cells. Comparison of LPS-treated and untreated Lifeact- EGFP labeled HoxB8 monocytes. (A) In contrast to LPS-treated HoxB8 monocytes, untreated control cells presented with no or few podosomes. The actin was diffusely distributed throughout the cell body with an enrichment at the cell membrane. Scale bar: 10 µm. (B) Traction force analyses reveal a big gap between both groups *($p<0.001$)* as calculated with the Mann-Whitney-U test.

Comment 7:
“Related to this, in several cases the horizontal stress occurs in areas of the cell that do not show podosomes; see e.g. Fig. 7C where podosomes are located towards the cell centre (in the area with large Uz) but horizontal stress (Fxy) is at the periphery and outside the podosome-rich area. If the cell forms no focal adhesion contact points in this podosome-free region, it is difficult to envision how the cell can apply force to the substrate here as the region would then be free of any anchor points of any type.”

Answer: We kindly thank Reviewer 2 for this comment. Originally, the figure was intended to emphasize the colocalization and, thus, the level to which our model could explain the observed traction hotspots. To our regret, the contrast appears to be too weak, leading to unintended misinterpretations. To this end, we now adjusted the brightness and global contrast (by

© 2022. Published by The Company of Biologists under the terms of the Creative Commons Attribution License (https://creativecommons.org/licenses/by/4.0/).

background subtraction) to highlight the colocalization in this example (Fig. 5C). To reduce signal overlaps, we also separated the Lifeact signal from Uz. Now, the two pieces of information regarding our model are better visualized. The traction ring covers the border of the podosome cluster, within which, as Reviewer 2 points out, the vast majority of podosomes are located.

Comment 8:
"In Fig. 6, the authors discuss a "bending model" and a "buckling model". It is concluded that the buckling model is more probable and that the buckling occurs due to a contractile network on the ventral side of the cell. It remains somewhat unclear to which extent this network is tied to the presence of podosomes. In my view, the observed stress pattern is not specific to monocytes or podosomes, but similar patterns are in fact also observed for other cells and for focal adhesion contacts."

Answer: We thank Reviewer 2 for pointing out the importance of the mechanical patterns' context. Indeed, as we use well-established methods on single cells, we get results that share many similarities to previously observed reports. However, in contrast to most other cell types, our cells display podosome clusters and lack stress fibers, which are the classical structures used to explain cellular force generation. Furthermore, we now add more experimental data that directly supports the buckling model by comparing the nuclear position with the indentation. As illustrated in Fig. 4A, the z indentation is correlated with the nuclear position in the bending model. However, we find that the z force centers and the nuclear centers do not overlap in our cells (Fig. 4E), whereas the z force centers and cell centers do.
To improve the paper, we now also mention that similar mechanisms might play a role in other cell types.

"*Minor points:*"
Comment 9:
"Fig. 2C shows a histogram of observed deformation in Z and claims to highlight the lower and upper limit of the 95% interval. However, these limits are themselves shown asintervals (grey areas). It remains unclear why these are regions rather than lines."

Answer: We adapted former Fig. 2C (now Fig. S1F). The originally shaded area was meant to emphasize the uncertainty of the percentiles across gels. We now show the 2.5th and 97.5th percentiles across several gel regions (n = 12 from four independent experiments) in two separate boxplots. Therefore, it should be less confusing and still illustrate the uncertainty between different gels and locations.

Comment 10:
"Fig. 2D shows displacement fields (Ux, Uy, Uz) and the traction stress obtained from these (Txy and Tz). The panel also shows a further illustration of "stress". It remains unclear what is meant by this and how "stress" would differ from Txy and Tz. Please explain/clarify."

Answer: We kindly thank Reviewer 2 for commenting on this need for clarification. Unfortunately, the mechanical terms used in the field are not entirely consistent. Therefore, we now defined the terms used in the manuscript more thoroughly, to make them most comparable to others and consistent in the manuscript. Briefly, *traction stress* (or *traction*) is defined as the directional force component per area and carries the unit Pascal. The normal component of the stress that is generated inside a material (i.e., without being exerted externally) and arises from mediating exerted tractions (*internal normal stress*) is called *internal stress* in this study. As it is also a stress, it carries the unit Pascal. However, high internal stress only reflects the forces transmitted within the cell. As soon as the material is locally coupled to the substrate, tractions are exerted while the stress locally vanishes. Here we noticed the potential application for the interpodosomal network.

Comment 11:
"Fig. 3A claims that the "captured time interval is 5s", although the scale in the plot shows a 16min x-axis. Clarify and correct if necessary. While the time lapse images in Fig. 3A are described in the caption, nothing is said about the plot at the bottom of Fig. 3A. Please expand the caption."

© 2022. Published by The Company of Biologists under the terms of the Creative Commons Attribution License (https://creativecommons.org/licenses/by/4.0/).

Answer: We thank the reviewer for the hint about the wording. As we removed the S100A8/A9 part, the original figure was changed to figure 2 and does not contain a time series anymore.

Comment 12:
"Fig. 3B lacks information on the number of observations/samples/podosomes used to generate the boxplots. In addition, it would be clearer to show the image first, followed by the boxplot, rather than the other way around."

Answer: We thank Reviewer 2 for this comment and added the sample sizes in the revised manuscript. The order was changed to make the new figure more readable, as suggested (Fig. 2F).

Comment 13:
"The caption to Fig. 4A seems poorly written ("Though without correlation, traction peaks…")."

Answer: We agree with the reviewer, and the phrase does not appear in the new figure (Fig. 2).

Comment 14:
"In contrast to earlier figures, Fig. 4B discusses Uz (vertical displacement) rather than Tz (vertical stress). Wouldn't it be more relevant to the mechanical analysis to discuss vertical stress (and to consistently analyse one of both throughout the manuscript)?"

Answer: We agree with the reviewer that the definitions of these variables and their use was discussed too briefly. Though mechanically interesting, we only use out-of- plane/vertical tractions (Tz) to compute the ratio between in-plane and out-of-plane strain energies (Ez is equivalent to Tz multiplied by Uz for each coordinate) as well as the z force center (Fig. 4E). Instead, we focus on the out-of-plane displacement (Uz), which reflects the substrate indenting protrusion throughout the study. We initially decided on protrusion (Uz) because it is a more easily understood phenomenon than vertical traction: While the latter is the cause, the former represents the consequence, i.e., the movement/bulging into the substrate, which is easier to imagine. Finally, our mathematical model depends on the parameter *h* (Fig. 5B), which represents the maximal indentation. We considered our model to be easier to follow if the parameters could be derived from our illustrated observations. Therefore, the compressive force (Txy) and radii (*a*) were visualized by the rotational projection of Txy (Fig 2H) and *h* by the rotational projection of Uz, respectively. To reduce confusion and misinterpretation, we only visualize Tz in the revised manuscript to illustrate the force center relation (Fig. 4E).

Reviewer #3

Advance Summary and Potential Significance to Field:

"Using lifeact expressing HoxB8 derived monocytes and 2,5D force microscopy, the authors address an interesting aspect of podosome functionality. They find that the indentation a cell imposes on the substrate is not explained by single podosome pushing forces but rather by a collectively generated mechanism that seems to result from the traction forces between podosomes. Two principle models could explain this effect: the nucleus being pushed into the substrate (bending model) or buckling forces arising from interpodosomal contractile forces (buckling model). Including a simple mechanical model, the authors argue for buckling. This seems to be thorough work and the result of deep mechanobiological considerations. The question is also interesting and if true, this is certainly an interesting aspect of podosome biology. "

Comments for the Author:

"Major points:"
Comment 1:
"I have a major problem with this manuscript as I find the writing largely incomprehensible. Passages read like a word by word translation of german into english and the whole terminology is way more complicated than necessary. I spent quite some time with the manuscript and only understood fractions. I doubt that a less motivated reader will make it very far. Presumably most

© 2022. Published by The Company of Biologists under the terms of the Creative Commons Attribution License (https://creativecommons.org/licenses/by/4.0/).

cell biologists will stop reading with the last sentence of the abstract, which is totally cryptic and therefore representative for the rest of the text. I personally think the text would benefit a lot from shortening 50% and simplifying language with the help of a native speaker."

Answer: We are sorry that our text was hard to read, and we are thankful for the direct and open criticism. To improve, we now largely revised the manuscript with a focus on the language. Additionally, we tried to shorten many parts, although we could not reach the suggested 50% reduction, mainly because we had included new experiments that were requested during the review process. Additionally, we had the paper checked by a native English speaker to avoid unclear language.

Comment 2:
"I also have a problem with the very indirect way of distinguishing between the bending and buckling model. The key here seems to be the nucleus and I do not see why this could not be directly addressed with experimentation. The authors could analyse enucleated cells or ventral areas away from the nucleus. If indentation happens without the nucleus, this would strengthen the buckling model enormously. Analysing nuclear shape in relation to the indentation could also be informative."

Answer: We agree with Reviewer 3 that nuclear analyses further contribute to the study. As we did not manage to generate enucleated cells, we focused on quantifying forces and nuclear position. To this end, we performed additional experiments in which mechanical measurements were complemented by nuclear staining using Hoechst 33342 at a low concentration of 0.1 µg/mL to minimize mechanical alterations (Fig. 4C- E). First, we compared the size of the nucleus and the indented regions as well as the Txy force rings and the indentation region Uz (Fig. 4C). This comparison showed no significant difference between the Txy and Uz slope diameters (*p =0.18*), as expected by their moderate correlation (Fig. 4B). However, we did find that the nucleus is about three times smaller than the indentation region. Notably, this does not rule out bending but strengthens the idea of a less nucleus-dependent indentation. To directly challenge the two models, we analyzed the Hoechst-stained cells with respect to a mutually exclusive assumption derived from bending and buckling theory: Bending theory assigns the cause of indentation to the nucleus and, thus, demands that the z force center (i.e., the center of mass of the weighted z force map) overlaps with the nucleus' center of mass (COM). In contrast, buckling assumes the indentation to be caused by the network and, thus, demands the z force center to overlap with the cell's COM. We observed the closest distance between the cell center and the z force center among all cells (mean ± s.e.m.: 1.6 ± 0.1 µm vs 3.7 ± 0.2 µm, *p < 0.001*) (Fig. 4E). Another way to study the relation between nucleus and indention is by quantifying the distance between the nucleus to the actual force center (NF) and the COM to the z-force center (CF). A ratio of these NF/CF distances with a value larger than 1 means that the nucleus is further away from the force than the cell center, which is consistent with the buckling model. In contrast, a value smaller than 1 supports the bending model. We find here a value of 3.1 ± 0.3 (mean ± s.e.m.), which, hence, supports the buckling model (one-sample t-test: p-value < 0.001).

Comment 3:
"Another problem I had was the distinction the authors make between cortical actin and the interpodosomal actin network. In my eyes these are either tightly interlinked structures, or possible even the same structure. The authors argue that these are two separate networks and I cannot see the evidence for this."

Answer: We agree with Reviewer 3 in that this separation is confusing. We had two networks in mind to better illustrate that contraction can happen between the podosomes, but it can also happen on the cortex level. In the revised version, we no longer make this distinction, as it is not required.

Comment 4:
"Another set of experiments that could be very informative is: imaging the actin network together with force microscopy and then pharmacologically disassembling actin and myosin. The spatio-temporal correlation between a gradually disassembling acto-myosin system and the relaxation of the substrate could support either one or the other model."

© 2022. Published by The Company of Biologists under the terms of the Creative Commons Attribution License (https://creativecommons.org/licenses/by/4.0/).

Answer: We thank the reviewer for the suggestion! Indeed, we can see such switches when looking the correlation of the gradually disassembling actomyosin network, we find the predicted critical values (Fig. S2J). We followed the suggested experiments by continuously imaging cells after addition of cytochalasin D (2 µM) at an interval of 13 seconds over around 24 minutes (Fig. S2D+H-J). The substrate beneath the cells relaxes due to cytochalasin D (Fig. 4H-J) and indeed reflects the reverse event shown in former Fig. 6E (now Fig. S2C+E-G) regarding indentation geometry. In particular, the broad indentation slowly disappears (Fig. S2I). With respect to mechanics, we plotted the maximal internal stress against the respective local mean indentation (4 µm x 4 µm). While the bending model predicts an entirely linear relation, the buckling model predicts a kink due to reaching the buckling threshold. Indeed, below about 100 Pascal the indentation does not appear to increase despite an increase in stress, but then it suddenly starts to rise linearly (Fig. S2G+J).

References:

Adlerz, K. M., Aranda-Espinoza, H. and Hayenga, H. N. (2015). Substrate elasticity regulates the behavior of human monocyte-derived macrophages. *Eur. Biophys. J. 2015 454* **45**, 301–309.

Andreu, I., Falcones, B., Hurst, S., Chahare, N., Quiroga, X., Le Roux, A. L., Kechagia, Z., Beedle, A. E. M., Elosegui-Artola, A., Trepat, X., et al. (2021). The force loading rate drives cell mechanosensing through both reinforcement and cytoskeletal softening. *Nat. Commun. 2021 121* **12**, 1–12.

Balaban, N. Q., Schwarz, U. S., Riveline, D., Goichberg, P., Tzur, G., Sabanay, I., Mahalu, D., Safran, S., Bershadsky, A., Addadi, L., et al. (2001). Force and focal adhesion assembly: A close relationship studied using elastic micropatterned substrates. *Nat. Cell Biol.* **3**, 466–472.

Blakney, A. K., Swartzlander, M. D. and Bryant, S. J. (2012). The effects of substrate stiffness on the in vitro activation of macrophages and in vivo host response to poly(ethylene glycol)-based hydrogels. *J. Biomed. Mater. Res. A* **100**, 1375.

Haley, K. E., Kronenberg, N. M., Liehm, P., Elshani, M., Bell, C., Harrison, D. J., Gather, M. C. and Reynolds, P. A. (2018). Podocyte injury elicits loss and recovery of cellular forces. *Sci. Adv.* **4**, 8030–8057.

Jones, G. E., Allen, W. E. and Ridley, A. J. (1998). The Rho GTPases in Macrophage Motility and Chemotaxis. *Cell Adhes. Commun.* **6**, 237–245.

Kronenberg, N. M., Liehm, P., Steude, A., Knipper, J. A., Borger, J. G., Scarcelli, G., Franze, K., Powis, S. J. and Gather, M. C. (2017). Long-term imaging of cellular forces with high precision by elastic resonator interference stress microscopy. *Nat. Cell Biol.* **19**, 864–872.

Labernadie, A., Bouissou, A., Delobelle, P., Balor, S., Voituriez, R., Proag, A., Fourquaux, I., Thibault, C., Vieu, C., Poincloux, R., et al. (2014). Protrusion force microscopy reveals oscillatory force generation and mechanosensing activity of human macrophage podosomes. *Nat. Commun.* **5**, 1–10.

Legant, W. R., Choi, C. K., Miller, J. S., Shao, L., Gao, L., Betzig, E. and Chen, C. S. (2013). Multidimensional traction force microscopy reveals out-of-plane rotational moments about focal adhesions. *Proc. Natl. Acad. Sci. U. S. A.* **110**, 881-886.

Lehtimäki, J. I., Rajakylä, E. K., Tojkander, S. and Lappalainen, P. (2021). Generation of stress fibers through myosin-driven reorganization of the actin cortex. *Elife* **10**, 1–43.

Meddens, M. B. M., Pandzic, E., Slotman, J. A., Guillet, D., Joosten, B., Mennens, S., Paardekooper, L. M., Houtsmuller, A. B., Van Den Dries, K., Wiseman, P. W., et al. (2016). Actomyosin-dependent dynamic spatial patterns of cytoskeletal components drive mesoscale podosome organization. *Nat. Commun.* **7**, 1–17.

Proag, A., Bouissou, A., Mangeat, T., Voituriez, R., Delobelle, P., Thibault, C., Vieu, C., Maridonneau-Parini, I. and Poincloux, R. (2015). Working Together: Spatial Synchrony in the Force and Actin Dynamics of Podosome First Neighbors. *ACS Nano* **9**, 3800–3813.

Sabass, B., Gardel, M. L., Waterman, C. M. and Schwarz, U. S. (2008). High Resolution Traction Force Microscopy Based on Experimental and Computational Advances. *Biophys. J.* **94**, 207.

Tambe, D. T., Croutelle, U., Trepat, X., Park, C. Y., Kim, J. H., Millet, E., Butler, J. P. and Fredberg, J. J. (2013). Monolayer Stress Microscopy: Limitations, Artifacts, and Accuracy of Recovered Intercellular Stresses. *PLoS One* **8**, e55172.

© 2022. Published by The Company of Biologists under the terms of the Creative Commons Attribution License (https://creativecommons.org/licenses/by/4.0/).

Second decision letter

MS ID#: JOCES/2021/259042

MS TITLE: 2.5D tractions in monocytes reveal mesoscale mechanics of podosomes during substrate indenting cell protrusion

AUTHORS: Hendrik Schürmann, Fatemeh Abbasi, Antonella Russo, Arne D. Hofemeier, Matthias Brandt, Johannes Roth, Thomas Vogl, and Timo Betz
ARTICLE TYPE: Research Article

We have now reached a decision on the above revised manuscript.

To see the reviewers' reports and a copy of this decision letter, please go to: https://submit-jcs.biologists.org and click on the 'Manuscripts with Decisions' queue in the Author Area. (Corresponding author only has access to reviews.)

As you will see, reviewer #3 is satisfied with your revisions and recommends publication. However, reviewer #1 still has some issues that you will need to address before submitting your final manuscript. These concern the determination of the traction forces in Fig. 2E, the interpretation of the results in Fig. 3, and a statement in the discussion. I trust that you will be able to properly deal with them and look forward to receiving a further revision of your paper.

Please ensure that you clearly highlight all changes made in the revised manuscript. Please avoid using 'Tracked changes' in Word files as these are lost in PDF conversion.

I should be grateful if you would also provide a point-by-point response detailing how you have dealt with the points raised by the reviewers in the 'Response to Reviewers' box. Please attend to all of the reviewers' comments. If you do not agree with any of their criticisms or suggestions please explain clearly why this is so.

Reviewer 1

Advance summary and potential significance to field

Advance made is comparable to the original manuscript.

Comments for the author

In this revised manuscript by Schurmann et al., several adjustments have been made with respect to the original submission that make the data more robust and the overall readability of the manuscript better. Also some chemical perturbations are added to provide some mechanistic insight into the origin of the traction forces. Although these changes have improved the overall quality of this manuscript I still have some major concerns related to the data and its interpretation by the authors. Also, while the overall readability is better than the original manuscript, still some concepts seem to mixed up and even for an expert, I find it hard to get through.

- In the text of the revised manuscript, it is now stated that Ez/Exy is 0.29 ± 0.16 while in the original manuscript it was about 0.1 (as extracted from the box plot in original Fig. 3D). What is the reason for this discrepancy?
Also, it seems like this value is calculated on a total cell level. If indeed so, what does this value then mean and how should it be interpreted with respect to single/collective podosome behavior?
- The relationship and interpretation of the cluster-wide protrusions and the individual protrusion both in xy as well as in z are still unclear. The authors now show some nice traction force and indentation maps for the xy and z forces (Fig. 2E). Yet, it is unclear how the Txy at the single podosome level should be interpreted. If there are indeed in-plane tractions underneath a

© 2022. Published by The Company of Biologists under the terms of the Creative Commons Attribution License (https://creativecommons.org/licenses/by/4.0/).

single podosome, how are these created? What molecular mechanism do the authors propose for this?

- For 2F, similar as in the original manuscript, the spatial relationship between the traction forces (xy) and podosomes is quantified and on the basis of this, the authors conclude that “no mutual colocalization between traction hot spots and podosomes”. Yet, it is unclear how the traction force peaks are determined. From the traction image (Fig. 2E), it seems like many more traction force peaks are present within the cell. Also, if the method used by the authors can by definition only measure traction with a minimal distance of 4um, then I wonder how this quantification would result in a useful interpretation of the data.
- Although the authors have addressed my comment why the overall traction forces seem to increase, the interpretation of the results in Figure 3 is still unclear. It seems that even locally, there is a correlation between Txy and Uz (which is actually also apparent in Fig. 2E). At the cell level, the buckling mechanism may explain this, but the authors do not provide a proper explanation for this apparent local correlation. Also, the authors state that “the mechanical patterns…did not show any substantial changes in topography”. While this may be true for the major forces on the periphery of the cell, there seem to be a lot of changes in the intracellular patterns. Do these correlate with the formation of individual podosomes? Also, what molecular mechanism explains the presence of constant traction peaks in the periphery? Although It seems like the authors point towards the buckling model as an explanation for this phenomenon, still some fixed “peripheral contact points” have to exist for these traction forces to be stable. Yet, no evidence is provided for this. Can the authors show that podosomes at those traction peaks are more stable than others?
- At the end of the results the authors state that “Therefore, the out-of-plane deformation also is likely to be caused by cluster effects.” This again is a statement that is not convincingly shown by the authors and adds to the confusion of how to interpret the in-plane and out-of-plane deformations. It seems like the authors implicitly conclude that single podosomes cannot exert out-of-plane deformation? What evidence do the authors have for this statement?

<u>Reviewer 3</u>

Advance summary and potential significance to field

The authors responded competently and adequately to all my concerns and I think the manuscript improved a lot. This is important and scientifically very sound work. The manuscript is still not the easiest read and maybe for a final version the authors could still put some work into making the paper more accessible for the general cell biologist. One suggestion would be to have a very simple and clear summary sentence at the end of each results section.
Saying this, I support publication of the manuscript as it stands.

Comments for the author

The authors responded competently and adequately to all my concerns and I think the manuscript improved a lot. This is important and scientifically very sound work. The manuscript is still not the easiest read and maybe for a final version the authors could still put some work into making the paper more accessible for the general cell biologist. One suggestion would be to have a very simple and clear summary sentence at the end of each results section.
Saying this, I support publication of the manuscript as it stands.

Second revision

<u>Author response to reviewers' comments</u>

We would like to thank the editor and the reviewers for their time in reading and evaluating our revised version. We were able to address all raised points. Explicitly, we would like to state that our work does not contradict in any moment the pushing forces of single podosomes. We point this out at several parts of the paper. We are fully convinced that the pushing forces of single podosomes are important, and we here only provide an additional mechanism that helps indenting the substrate on the full cell level. As you will see in the detailed answers, a series of concerns can be easy relieved by pointing out that single podosome forces are

© 2022. Published by The Company of Biologists under the terms of the Creative Commons Attribution License (https://creativecommons.org/licenses/by/4.0/).

of course important.

We hope that the here provided replies are clear and look forward to hear your opinion.

Reviewer #1

Advance Summary and Potential Significance to Field:

"Advance made is comparable to the original manuscript."

Comments for the Author:

Comment 1:
"In this revised manuscript by Schurmann et al., several adjustments have been made with respect to the original submission that make the data more robust and the overall readability of the manuscript better. Also some chemical perturbations are added to provide some mechanistic insight into the origin of the traction forces. Although these changes have improved the overall quality of this manuscript I still have some major concerns related to the data and its interpretation by the authors. Also, while the overall readability is better than the original manuscript, still some concepts seem to mixed up and even for an expert, I find it hard to get through."

Answer: We are glad that the changes were to the reviewer's overall liking, and we respond to the raised concerns point-by-point in the following.

Comment 2:
"In the text of the revised manuscript, it is now stated that Ez/Exy is 0.29 ± 0.16 while in the original manuscript it was about 0.1 (as extracted from the box plot in original Fig. 3D). What is the reason for this discrepancy? "

Answer: The reviewer has very impressively spotted here an error that we had in the plotting script of the first manuscript. Specifically, "uz" instead of "fz" was referred to during one assignment prior to calculation of the out-of-plane strain energy (Ez). It was only calculated for this figure without any reference later, and by nature of the close relation between uz and fz exposed no direct plausibility error. This error was already fixed when reanalyzing the data during the revision, and hence the correct value of
0.29 ± 0.16 is now reported.

Comment 3:
"Also, it seems like this value is calculated on a total cell level. If indeed so, what does this value then mean and how should it be interpreted with respect to single/collective podosome behavior?"

Answer: The Ez/Exy ratios were calculated on the cell-scale across the podosome cluster bearing cells. At this initial part of the manuscript, we did not yet relate 2.5D cell-scale mechanics to podosomes, but first introduced podosomes (Fig. 2A+B) and mechanics (Fig. 2C) separately. As our manuscript does not address single podosomes, but has a focus on the collective effect as we directly state in Lines 23-24 ("Complementary to previous studies focusing on individual podosomes, here we investigated cell-wide force generation […]"), we compare in-plane and out-of-plane mechanics on the cell level as pointed out in Lines 174-176 ("highlighting that a substantial proportion of the deformation energy generated by the cell was being expended in the z direction"). We here demonstrate that across all these cells approximately a quarter of the strain energy is spent in z. Hence, Ez on the whole cell level may fulfill a biological purpose, additionally to the well described and studied z- forces generated by single podosomes. However, here we do not infer any information on the single podosome level.

Comment 4:
"The relationship and interpretation of the cluster-wide protrusions and the individual protrusion both in xy as well as in z are still unclear. The authors now show some nice

© 2022. Published by The Company of Biologists under the terms of the Creative Commons Attribution License (https://creativecommons.org/licenses/by/4.0/).

traction force and indentation maps for the xy and z forces (Fig. 2E). Yet, it is unclear how the Txy at the single podosome level should be interpreted. If there are indeed in-plane tractions underneath a single podosome, how are these created? What molecular mechanism do the authors propose for this?"

Answer: The presence of focal in-plane tractions (Txy) within the broad protrusive area cannot be explained by current mechanical models, as these aim at describing the z- forces of single podosomes. Our data additionally suggest these tractions to occur at podosomes, which appears to conflict with the single podosome protrusion model (Bouissou et al., 2017). However, in previous work the lateral (xy) forces were not addressed and hence not integrated in these models. Accordingly, we understand the reviewer's comment as question if and how our model may explain these in-plane tractions.
Based on the proposed network model, in-plane tractions are assumed to be transmitted at individual podosomes (i.e., network nodes) if two conditions are met: (1) the respective podosome is adherent to the substrate, and (2) the net force at the podosome due to network contraction is not equal to zero (Fig. 5A *red*). The first condition is a hallmark of podosome function and mediated by integrins (Linder and Aepfelbacher, 2003). According to our model, the second condition is assumed to be generally met for peripherally located podosomes, which cannot relay the centripetal contraction force component to neighbored podosomes. Instead, it is transmitted to the substrate and results in the observed "constant" centripetal in-plane traction component (Fig. 2G). The molecular basis is an actomyosin network that connects the nodes. As this is a dynamic network, it implies the temporary occurrence of imbalance on the single podosomes, which leads to the low number of central in-plane tractions. As these central in-plane tractions are usually transient (Fig. 2G), it suggests that the network's connectivity is rapidly reestablished. Conveniently, our model can explain the "constant" (peripheral) and "transient" (central) tractions by one mechanism. As we only require a lateral coupling between podosomes via the actomyosin network, our model is not conflicting with previous models, but rather extends these.

<u>*Comment 5:*</u>
"For 2F, similar as in the original manuscript, the spatial relationship between the traction forces (xy) and podosomes is quantified and on the basis of this, the authors conclude that "no mutual colocalization between traction hot spots and podosomes". Yet, it is unclear how the traction force peaks are determined. From the traction image (Fig. 2E), it seems like many more traction force peaks are present within the cell."

Answer: The reviewer highlights that local signal peaks of the traction map become apparent in Fig. 2 that we did not include into our spatial analysis between podosomes and traction signal peaks (Fig. 2F).
Local traction signal peaks do not necessarily represent real tractions in TFM. Although we employ regularization to reduce noise, circumscribed traction signal peaks of various magnitude appear in cell-free areas across all measurements (e.g. Fig. 2D+E+G, Fig. 3A). We must assume that this noisy pattern also occurs within the cell and superimposes but do not represent real tractions. Therefore, thresholding based on the background signal is mandatory for technical reasons. The peak detection was then designed as explained in the method section (Lines 639-645). Based on the noise in our study, we empirically defined the lowest threshold as 50 Pascal to avoid potential overestimates in weak cells. In the revised version, this is now mentioned more prominently to be as accurate as possible (Line 642-643). The empirical threshold is hence not arbitrary but based on a careful quantification of the background noise levels. Additionally, we interpreted the results in the context of these limitations. What we finally claim is largely independent of the exact individual in-plane tractions and the exact thresholds: (1) the podosome cluster spans over the entire area defined by the centripetal in-plane tractions, and (2) at some but not all podosomes we see transmission of in-plane tractions.

<u>*Comment 6:*</u>
"Also, if the method used by the authors can by definition only measure traction with a minimal distance of 4um, then I wonder how this quantification would result in a useful interpretation of the data."

Answer: As mentioned in the manuscript (Lines 657-679), we employed point discrimination, i.e.,

© 2022. Published by The Company of Biologists under the terms of the Creative Commons Attribution License (https://creativecommons.org/licenses/by/4.0/).

the capability to distinguish closely located force centers, to define resolution. Therefore, the correct interpretation is that for xy-tractions, traction centers with distances up to approximately 2 µm can be distinguished by our method (Lines 141-143). For indentations, the resolution limit ranges lower at approximately 4 µm (Lines 145-146) as mentioned by the reviewer. However, we did not analyze individual indentation peaks for this reason, but only discuss here the in-plane tractions Of note, tractions can still be measured below the resolution limit, however, the number of underlying force centers cannot be determined. Although we would be happy to see future data at higher resolution, for this study we consider the lateral resolution of 2 µm for in-plane tractions as sufficient. Practical evidence for this statement can be found in Fig. 2E: If the resolution limit would be unsuitable for our purpose, we would be unable to observe multiple individual traction foci that can be even visually linked to local actin reorganization. Instead, we would observe a smeared-out ring.
In conclusion, the resolution for lateral traction is 2 µm, and the data in Fig. 2E demonstrates that the resolution is sufficient to locate force centers for the presented distance measurements.

<u>*Comment 7:*</u>
“Although the authors have addressed my comment why the overall traction forces seem to increase, the interpretation of the results in Figure 3 is still unclear. It seems that even locally, there is a correlation between Txy and Uz (which is actually also apparent in Fig. 2E). At the cell level, the buckling mechanism may explain this, but the authors do not provide a proper explanation for this apparent local correlation.“

Answer: The reviewer raises an interesting point, namely if there is also a local correlation between Txy and Ux. As mentioned in the paper, we do not aim to study the effect of individual podosomes. We agree with the reviewer that this might be an interesting question, however it has already been very well addressed previously. Such potential local correlations do not contradict the cell scale model, as the individual podosomes can of course provide additional indentation forces as known from previous work. However, in this study we focus on the cell level indentation and our network based buckling model only adds to these previous explanations. In short, the reviewer is absolutely right that this is an interesting possibility, but including such investigation is not the aim of the paper, would in our opinion confuse the reader and would recapitulate previous findings. Given the low resolution for the indentation, we do not feel that our experiment is optimized to address such question, which is the reason why we focus on the question on the cell level, which we can address well.
To improve the paper, we now add two short sentences to point this out:
“It should be noted that dynamic lateral and indentation force centers in the inner part of the cell can be attributed to previously described and well-known local forces generated by podosome. However, the cell scale indentation as observed here seems to be an additional effect that can be explained as a global cell-level effect” (Lines 293- 297).

<u>*Comment 8:*</u>
“Also, the authors state that “the mechanical patterns…did not show any substantial changes in topography”. While this may be true for the major forces on the periphery of the cell, there seem to be a lot of changes in the intracellular patterns. Do these correlate with the formation of individual podosomes?“

Answer: Unfortunately, it is extremely difficult to distinguish the dynamic weak signals within the traction ring from background noise (see answer to comment 5). Hence, we have no substantial evidence of correlation between intracellular patterns and the formation of podosome. It would certainly be interesting to study the signals together with podosome formation. While this study focused on cluster-wide protrusions, a future study that focusses on the in-plane tractions may give an answer to this interesting question.

<u>*Comment 9:*</u>
“Also, what molecular mechanism explains the presence of constant traction peaks in the periphery? Although It seems like the authors point towards the buckling model as an explanation for this phenomenon, still some fixed “peripheral contact points” have to exist for these traction forces to be stable. Yet, no evidence is provided for this. Can the authors show that podosomes at those traction peaks are more stable than others?“

© 2022. Published by The Company of Biologists under the terms of the Creative Commons Attribution License (https://creativecommons.org/licenses/by/4.0/).

Answer: Our measurements were relatively brief with 16 minutes per cell to allow for dynamic measurements. Therefore, the "constant" we refer to can naturally only account for this period over which podosomes can be stable (Murphy and Courtneidge, 2011). The "fixed peripheral contact points" proposed by the reviewer are a possible explanation. Another explanation is that if one podosome loses contact locally, the network snaps a little bit back until a next nearby podosome takes over. Hence, given a sufficient number of podosomes at the periphery, there is no contradiction between dynamic podosomes on the local level, and stable forces at the cell level. We do not have the resolution to make any clear statement on this, hence we do not discuss it. Briefly, we do not show proof of higher stability, as this is not a requirement of our model.

Comment 10:
"At the end of the results the authors state that "Therefore, the out-of-plane deformation also is likely to be caused by cluster effects." This again is a statement that is not convincingly shown by the authors and adds to the confusion of how to interpret the in-plane and out-of-plane deformations. It seems like the authors implicitly conclude that single podosomes cannot exert out-of-plane deformation? What evidence do the authors have for this statement?"

Answer: We are sorry for this apparently misleading sentence and now added "broad, cell-wide" (Line 383) to emphasize the size-scale of our conclusion. We understand that the reviewer is concerned that our paper can be interpreted as argument against individual podosome forces. This is absolutely not our intention, and we mention this at several positions in the paper. As there is compelling evidence for single podosome protrusion (i.e., out-of-plane deformation) shown by PFM and ERISM (Kronenberg et al., 2017; Labernadie et al., 2014; Proag et al., 2015), we are convinced that individual podosomes exert out-of-plane forces. While our method is not optimized to study these individual forces (Lines 430-432), we highlight that the putatively buckling-induced broad indentation "acts in addition to the protrusion forces of individual podosomes" (Lines 34-35) and point out the complementary character of our findings (Lines 427-430).

Reviewer #3

Advance Summary and Potential Significance to Field, Comments for Author:

"The authors responded competently and adequately to all my concerns and I think the manuscript improved a lot. This is important and scientifically very sound work. The manuscript is still not the easiest read and maybe for a final version the authors could still put some work into making the paper more accessible for the general cell biologist. One suggestion would be to have a very simple and clear summarysentence at the end of each results section.
Saying this, I support publication of the manuscript as it stands."

Answer: We would like to thank the reviewer for the positive feedback of our manuscript and the constructive input during the first review round.
We gratefully adapted our manuscript according to the reviewer's suggestion and now included summary statements for the result section.

References:

Bouissou, A., Proag, A., Bourg, N., Pingris, K., Cabriel, C., Balor, S., Mangeat, T., Thibault, C., Vieu, C., Dupuis, G., et al. (2017). Podosome Force Generation Machinery: A Local Balance between Protrusion at the Core and Traction at the Ring. *ACS Nano* **11**, 4028–4040.

Kronenberg, N. M., Liehm, P., Steude, A., Knipper, J. A., Borger, J. G., Scarcelli, G., Franze, K., Powis, S. J. and Gather, M. C. (2017). Long-term imaging of cellular forces with high precision by elastic resonator interference stress microscopy. *Nat. Cell Biol.* **19**, 864–872.

Labernadie, A., Bouissou, A., Delobelle, P., Balor, S., Voituriez, R., Proag, A., Fourquaux, I., Thibault, C., Vieu, C., Poincloux, R., et al. (2014). Protrusion force microscopy reveals oscillatory force generation and mechanosensing activity of human macrophage podosomes. *Nat. Commun.* **5**, 1–10.

Linder, S. and Aepfelbacher, M. (2003). Podosomes: adhesion hot-spots of invasive cells. *Trends*

© 2022. Published by The Company of Biologists under the terms of the Creative Commons Attribution License (https://creativecommons.org/licenses/by/4.0/).

Cell Biol. **13**, 376-385.
Murphy, D. A. and Courtneidge, S. A. (2011). The "ins" and "outs" of podosomes and invadopodia: characteristics, formation and function. *Nat. Rev. Mol. Cell Biol. 2011 127* **12**, 413-426.

Proag, A., Bouissou, A., Mangeat, T., Voituriez, R., Delobelle, P., Thibault, C., Vieu, C., Maridonneau-Parini, I. and Poincloux, R. (2015). Working Together: Spatial Synchrony in the Force and Actin Dynamics of Podosome First Neighbors. *ACS Nano* **9**, 3800-3813.

Third decision letter

MS ID#: JOCES/2021/259042

MS TITLE: 2.5D tractions in monocytes reveal mesoscale mechanics of podosomes during substrate indenting cell protrusion

AUTHORS: Hendrik Schürmann, Fatemeh Abbasi, Antonella Russo, Arne D. Hofemeier, Matthias Brandt, Johannes Roth, Thomas Vogl, and Timo Betz
ARTICLE TYPE: Research Article

I am happy to tell you that your manuscript has been accepted for publication in Journal of Cell Science, pending standard ethics checks.

© 2022. Published by The Company of Biologists under the terms of the Creative Commons Attribution License (https://creativecommons.org/licenses/by/4.0/).
